# Sustaining corrected beliefs in false news headlines over time: The roles of correction format and recognizing corrections

**DOI:** 10.3758/s13421-025-01760-7

**Published:** 2025-08-06

**Authors:** Christopher N. Wahlheim, Paige L. Kemp, Robert W. Wiley, Andrew M. Engelhardt

**Affiliations:** 1 Department of Psychology, University of North Carolina at Greensboro, 296 Eberhart Building, PO Box 26170, Greensboro, NC 27402-6170 USA; 2 Department of Political Science, Stony Brook University, Stony Brook, NY USA

**Keywords:** Backfire effects, Belief updating, Fake news, False beliefs, Misinformation

## Abstract

Exposure to false information on social media can erode the accuracy of beliefs and memory. These effects can be mitigated by issuing fact-checked corrections. Mounting evidence suggests that corrections improve belief accuracy the most when consumers recognize that a statement was earlier corrected. Two experiments examined this relationship further by testing whether correction formats vary in how they promote encoding and recognition of corrections as well as belief accuracy. To assess baseline beliefs, participants first rated the veracity of true and false headlines from the internet. Participants then read three types of corrections that all included veracity labels. Corrections showed only false headlines from the prior phase, only true headlines that contradicted false headlines from the prior phase, or false and true headlines, in that order. Finally, participants rerated the veracity of the original headlines and rated their recognition that headlines were corrected, both immediately after the correction phase and again after 1 week (Experiment 1) or month (Experiment 2). Corrections improved belief accuracy by lowering veracity ratings for false headlines, especially for corrections with false and true information. Highly confident recognition of corrected headlines was associated with durable improvements in rbelief accuracy, whereas less confident recognition was associated with less improvement and sometimes impairment. Corrections with false and true information led to the most high-confidence recognition of corrections. Collectively, these findings suggest that modifying correction formats to promote encoding and recognition of corrections can lead to sustained improvements and belief accuracy over time that counteract regression to false beliefs.

**Supplementary Information:**

The online version contains supplementary material available at https://doi.org/10.3758/s13421-025-01760-7.

False information portrayed as true can be harmful. As one example, fake images of a missing Malaysia Airlines flight on social media led passengers’ families to believe the plane was found, causing them emotional distress (Boididou et al., 2018). As another example, belief in COVID-19 misinformation reduced vaccination intentions (Loomba et al., 2021), which could have contributed to negative health outcomes during the pandemic. One way to counteract such effects is to correct misinformation after the fact. Although corrections reduce false beliefs (for a review, see Porter & Wood, 2024), some formats are more effective than others (for reviews, see Ecker et al., 2022; Wittenberg & Berinsky, 2020). One variable that may determine the efficacy of corrections over time is how their format promotes encoding and subsequent recognition that a topic was associated with a correction. The present study examined how correction formats, with varying elements from social media displays, promote the recognition of corrections and how such recognition counteracts the regression of beliefs over time. The corrections here varied in whether they presented both false and true information, or only false or true information. The present study is relevant to the literatures on repetition and beliefs in true and false information, misinformation corrections, and the interdependence of memory and beliefs. We selectively summarize only the most relevant studies from those literatures before describing our approach.

## Repetition and beliefs in true and false information

The literature on repetition and beliefs in true and false information is relevant to the study of correction effects because the perceived veracity of information varies based on the frequency of exposure to that information. Given that corrections repeat some or all aspects of earlier-learned information, correction formats could have varying consequences for perceptions of veracity. The literature on this issue has established that repeating information increases perceived veracity regardless of actual veracity (for reviews and a meta-analysis, see Brashier & Marsh, 2020; Dechêne et al., 2010; Udry & Barber, 2024). This repetition-induced truth effect was first observed when repeating plausible yet obscure true and false trivia statements (Hasher et al., 1977). Although this effect is often shown using trivia statements, it also generalizes to stimuli such as text passages, marketing claims, and news headlines (for a review, see Henderson et al., 2022). Regarding the latter, repeating false news headlines in a Facebook format, even when repetitions were labeled as false, led to increases in the perceived veracity of the headlines on a later occasion (Pennycook et al., 2018). Despite the pervasiveness of this effect, methods that direct attention to veracity can reduce or eliminate it (Brashier et al., 2020; Calvillo & Harris, 2023; Jalbert et al., 2020). This is promising for corrections that repeat full or partial content from false headlines to directly or indirectly guide attention to veracity.

The potential role of repetition-induced truth in the efficacy of correction formats may also be surmised by considering the mechanisms proposed to underlie the effect (for a review, see Unkelbach et al., 2019). The most relevant here are the interrelated frequency, recognition, familiarity, and processing fluency mechanisms. The original explanation offered for repetition-induced truth was that participants misattributed frequency information for veracity (Hasher et al., 1977), which was later shown to require overt recognition that information was repeated (Bacon, 1979). Building on these ideas, a dual-process perspective proposed that the feeling of familiarity induced by repetitions could be misattributed as truth unless consciously controlled recollection of contextual details, such as source credibility, overrode that nondiagnostic basis for perceived veracity (Begg et al., 1992; Fazio et al., 2015). This feeling of familiarity was conceived as fluent processing that could be misattributed to truth (Reber & Schwarz, 1999) unless the conditions led participants to associate fluency with falsity (Unkelbach, 2007).

From these perspectives, repeating elements from headlines with false information during corrections could have two consequences. Increasing the fluency of false information, either by showing it again or triggering its retrieval, could impair belief accuracy if the fluency is misattributed as truth. Conversely, improving memory for false details as such could provide a diagnostic basis for overriding familiarity and judging the information as false. This overarching view suggests that correction formats promoting elaborative encoding that leads to high-quality memories that include veracity information from corrections should lead to enhanced accuracy in the rating of headlines’ veracity. We develop this argument in the context of the present study after reviewing key studies of correction effects—some of which incorporate this perspective.

## Misinformation corrections

Predictions about the consequences of repeating false information during corrections can also be derived from studies of misinformation correction effects on various aspects of cognition. Correction effects have been studied in several paradigms that include the same key procedural aspects: False information is first learned and then corrected. Later, in a postcorrection phase, the effects of corrections on the constructs of interest (e.g., beliefs) are measured. Correction effects have been thoroughly documented in studies of the continued influence effect (for reviews and a meta-analysis, see Ecker et al., 2022; Lewandowsky et al., 2012; Walter & Tukachinsky, 2020). One task entails participants reading a narrative with false information that is retracted or refuted in a second narrative, then answering questions about the narrative events that could elicit inferences based on false or true information (Johnson & Seifert, 1998; Wilkes & Leatherbarrow, 1988). False information exerts a continued influence after corrections when inferences about narrative events are still based on that information. The magnitude of this effect varies with manipulations such as the frequency of information (Ecker et al., 2011), source credibility (Guillory & Geraci, 2013), correction timing (Rich & Zaragoza, 2020), and willingness to accept retractions (O’Rear & Radvansky, 2020), to name a few. Importantly, this effect also generalizes to stimuli such as real-world news (Lewandowsky et al., 2005).

Many accounts have attempted to explain the continued influence effect (for a review, see Ecker et al., 2022). However, we focus on those most relevant to the objective of the present study—namely, to characterize the cognitive mechanisms that determine how correction formats affect beliefs in false headlines and memory for those headlines. The selective retrieval account is a dual-process perspective which proposes that retractions add veracity information to false information, and that the type of retrieval processes engaged later determine the extent to which false information continues to have an influence (Ecker et al., 2011). Accordingly, when participants recollect contextual details, such as veracity information, false information will be less influential. Conversely, when retrieval is based on automatic familiarity, contextual details are not recollected, and false information will continue to have an influence. A related but more extreme perspective is the familiarity backfire account, which proposes that repeating false information can increase its perceived veracity above initial beliefs when consumers do not recollect contextual information diagnostic of veracity (Schwarz et al., 2007; Skurnik et al., 2005). However, rigorous studies have failed to observe the effect under appropriate conditions (Ecker et al., 2020; Prike et al., 2023; Swire-Thompson et al., 2020; Wood & Porter, 2019).

Alternatively, integration accounts converge on the proposal that the coactivation of false and true information can lead to memories that preserve the differences in information veracity. One variant of this account, the Knowledge Revision Components (KReC) framework (Kendeou et al., 2014, 2019), was proposed to explain how refutation texts that restate and correct false information affect memory for false and true information. The account proposes that the reactivation of encoded information allows it to become coactivated with updated information, which allows the updated information to become integrated and predominant over time. Another variant of this account, the Memory-for-Change (MFC) framework (Jacoby et al., 2015; Wahlheim & Jacoby, 2013), was proposed to explain how the episodic memory for an event could proactively facilitate or interfere with the memory for an event with shared and distinct features. The MFC framework shares the coactivation principle with the KReC framework in proposing that perceived events can reactivate and become associated with existing memories. However, the MFC framework incorporates the dual-process perspective by proposing that access to integrated memories requires recollection-based retrieval. Moreover, the account proposes that the reactivation of earlier memories while encoding updated events leads those memories to become more accessible. Consequently, when recollection fails during subsequent retrieval, the increased accessibility of outdated memories leads them to be more competitive.

Although these accounts are distinct frameworks, they mostly agree that the efficacy of sufficiently encoded corrections depends on whether consumers can retrieve contextual details that include veracity information. This perspective is relevant to the present study because the format of corrections should determine how veracity information is encoded and subsequently recollected. This point is supported by findings from studies of correction effects using different approaches. For example, in a study where participants rated their beliefs in myths and facts of unclear veracity before the myths were corrected, reductions in myth beliefs and inferences that indirectly measured beliefs were better sustained when recollection-based retrieval was more likely (i.e., at shorter retention intervals and for younger participants; Swire et al., 2017). Relatedly, in a study using the narrative paradigm, the influence of false information on inferences was lower for refutations that included complete reminders about earlier-read false information than for refutations with partial or no reminders (Ecker et al., 2017). Consistent with integration accounts, these findings suggest that repeating false information during corrections enabled coactivation that promoted recollection of information veracity. More generally, both studies point to an intricate relationship between memory and beliefs that may be critical for reducing the perceived veracity of false information and sustaining belief updating over time.

## Memory, beliefs, and their interdependence

Building on these ideas, more recent studies have examined how correction formats lead to differences in memory and belief updating. The research most relevant here examined how reminders and retrieval of false information during corrections affect memory for true information from corrections, memory for whether false information was corrected, and associations between the two (Kemp et al., 2022a, b, 2024b; Wahlheim et al., 2020). In contrast to the aforementioned paradigms that primarily focused on beliefs and inferences as indirect measures of beliefs, the memory and belief updating studies examined correction effects on cued recall or recognition and perceptions of the veracity (i.e., beliefs) of retrieved details. Thus, although the tasks allowed for inferences about the processes underlying memory retrieval, the procedures precluded assessment of potential familiarity-backfire effects, which can only be shown when beliefs are assessed for all of the studied stimuli. Another noteworthy difference is that the stimuli in the studies of memory and belief updating were news headlines from the internet that had been formally fact-checked.

As in the present approach, the memory and belief updating studies with reminder-based corrections conformed to a three-phase procedure. In Phase 1, participants first read true and false headlines of unclear veracity. In Phase 2, they read true headlines that affirmed true headlines and corrected false headlines from Phase 1. Some true headlines appeared after reminders of false headlines, and both included veracity labels (i.e., “correct” or “incorrect”). In Phase 3, participants attempted to recall (or recognize) true details, indicated their beliefs by rating the veracity of those details, indicated whether they remembered the topic being corrected in Phase 2, and if so, attempted to recall (or recognize) the corrected false detail. Regarding operational definitions, higher memory accuracy was shown by greater differences between correct retrieval of true information and incorrect retrieval of false information (i.e., intrusions), while higher belief accuracy was shown by greater differences between veracity ratings for correctly retrieved true details and incorrectly retrieved false details.

Reminder-based corrections that included veracity-labeled false and true information improved memory and belief accuracy compared with corrections with only true information (Kemp et al., 2022b; Wahlheim et al., 2020). Additionally, when false headlines appeared twice initially, reminders showing false details led to better memory accuracy than reminders that prompted recall of false details (Kemp et al., 2024b). However, the reverse was true when false information appeared once initially, suggesting that reminder efficacy depends on initial memory accessibility and cueing potential. Across studies, reminder-based corrections improved memory for corrections and recall of false details. This combination of memory outcomes indicating retrieval of contextual details was interpreted as showing that reminders enhanced encoding in a way that supported later recollection-based retrievals. Such retrievals were associated with improved memory accuracy in recall of true information and belief accuracy in assessments of the veracity of those retrievals. In contrast, evidence for proactive interference from exposure to false information was shown by lower recall of true information and more intrusions when corrections were not remembered, implicating a role for familiarity-based influences.

These findings of correction effects on memory and beliefs suggest that differences in belief updating partly depend on how corrections promote memory. However, no studies characterized such effects in a paradigm that assessed how belief changes are sustained over time, or whether repeating false information leads familiarity to backfire. Toward this goal, one study has examined the role of memory for corrections in sustaining belief change over time (Swire-Thompson et al., 2023). Participants rated their beliefs in true and false statements, then read corrections with the false and true information. Immediately and 1 month later, participants rerated their beliefs and rated their memory for whether information had been corrected or affirmed. Between-subjects associations showed that reduced beliefs in false statements regressed towards baseline levels to the extent that participants did not remember those statements being corrected. From immediate to delayed tests, beliefs never increased above the original ratings, showing that repeating false information did not lead to familiarity backfire. Taken with the studies of false headline corrections, these results suggest that belief updating following corrections of false headlines should be more pronounced and sustained to the extent that correction formats promote encoding and subsequent recognition of corrections. Moreover, although repeating false information during corrections could increase its perceived veracity, it may also promote associative encoding that supports later memory for veracity.

## The present study

The present study combined the approaches from the memory and belief updating paradigms to examine the roles of correction formats and recognition of corrections in belief change over time. Two experiments used variants of a task with features from paradigms assessing belief updating on immediate and delayed measures (e.g., Swire-Thompson et al., 2023) using social media presentation formats that increased the naturalism of the approach (for a similar approach, see Porter & Wood, 2022). Following prior work on repetition-induced truth (Pennycook et al., 2018), we presented news headlines instead of articles because people mostly read headlines on social media (Gabielkov et al., 2016). We designed the headline presentation format to resemble news headlines on social media, each with a statement below a related image. The paradigm included a first session with three phases and a second session with a fourth phase.

In Phase 1, participants read true and false headlines of unclear veracity and provided ratings of preexisting experience with the topics, which we refer to colloquially as “familiarity.” Next, participants rated the perceived veracity of the headlines, which we took to indicate their beliefs. The headlines included statements about real-world topics taken from reputable fact-checking websites. The familiarity ratings verified that participants had little experience with the news headlines. This allowed the outcome measures to be more sensitive to the effects of the manipulations and less influenced by existing knowledge.

In Phase 2, participants viewed corrections of the false information and affirmations of the true information from Phase 1. We used three veracity-labeled correction formats that varied in their inclusion of false and true details. One format included only repetitions of false details, and another included only presentations of true details that contradicted the false details. These conditions were included together to examine whether repeating false details would lead to a repetition-induced truth effect yielding higher veracity ratings for false headlines corrected with only false rather than only true details. A third format included corrections with false details that appeared before true details. This sequence was intended to resemble a situation where a social media consumer reads a fact-checked false headline and may then opt to read the corrective true details (i.e., a click-through correction). Integration accounts propose that this format will best promote associative encoding of true and false information, thus also promoting memory for corrections upon which perceptions of veracity can be made. Conversely, repetition-induced truth accounts predict that corrections repeating false information should lead to higher veracity ratings for false headlines than corrections with only true information. It should be noted that presenting false and true information leads to longer exposure time than corrections including only true or false information. Thus, viewing time could be used as a basis for belief and memory judgments. However, variants of this format increase cued recall of true details (e.g., Kemp et al., 2022b; Wahlheim et al., 2020), suggesting that integrative encoding should still play a role.

We tested these predictions by comparing veracity ratings for the same false headlines before and after corrections. In Phases 3 and 4, we also examined how correction formats affected recognition of corrections to determine the mnemonic contributions to belief accuracy. In Phase 3, which immediately followed corrections, participants rated the veracity of headlines and memory for the topic being corrected. In Phase 4, which occurred after 1 week or month, participants repeated the Phase 3 procedure. The rationale for extending the time intervening between Phases 3 and 4 from Experiments 1 and 2 was based on applied and theoretical considerations. For applied purposes, this allowed us to characterize how the formats differed in their efficacy over different time periods. For theoretical purposes, the perspective most relevant here is that recollection-based memory for contextual details, which include veracity labels, can promote accurate beliefs by providing diagnostic information that opposes repetition-induced truth. Extending the retention interval may reduce recollection thus allowing familiarity of the headline topics alone to exert a greater influence on veracity ratings (e.g., Swire et al., 2017).

## Hypotheses

Our first preregistered hypothesis pertains to correction effects on immediate belief updating. Corrections with labeled false and true information can improve recall of both details compared with corrections presenting only true information (Kemp et al., 2022b; Wahlheim et al., 2020). The benefits of corrections with both false and true information have also been observed for belief accuracy (Kemp et al., 2022b; Wahlheim et al., 2020). These findings are consistent with the view that presenting both details promotes their integration. Moreover, a pilot experiment using the first three phases of the present tasks showed greater immediate reductions in veracity ratings for false headlines after corrections that repeated false information, regardless of whether true information followed (see the OSF: https://osf.io/37vtp/). These findings led us to predict the same patterns of immediate reductions in the veracity ratings for false headlines across conditions, thereby showing improved belief accuracy, as opposed to repetition-induced truth. We did not have an a priori hypothesis about the durability of these belief changes over time.

Our second preregistered hypothesis pertains to the extent to which the manipulation of correction formats will lead to differences in recognition that false headlines were corrected. Note that we were most interested in the highest confidence correction recognition ratings under the assumption that such reports would be most likely to include instances of recollection-based retrieval. This assumption is based on the fact that “correction” recognition judgments, unlike standard probe recognition judgments, are responses indicating memory for contextual details—namely, veracity information that was provided during a specific temporal context (i.e., Phase 2). Based on behavioral and neural findings suggesting that source memory judgments primarily reflect recollection, especially for judgments held in high confidence (e.g., Thakral et al., 2015), we assumed that the high-confidence source judgments used in the present experiments would also primarily reflect recollection. However, to be certain, we do not assume that this measure is process pure (Jacoby, 1991) and acknowledge that source judgments can be made based on familiarity (Yonelinas, 1999). Notably, the design here rendered familiarity of little diagnostic value for source judgments: By including corrections and affirmations in Phase 2, familiarity was equated for those item types, thus requiring recollection to determine content veracity.

Before our pilot, no study had compared the memorability of corrections including only labeled false information, only labeled true information, and labeled false and true information. However, we have shown that memory for corrections that is most likely based on recollection is better for corrections with labeled false and true information than labeled true information alone (Kemp et al., 2022b). Consistent with these findings, our pilot results showed that memory for corrections was greatest for corrections with false and true information, intermediate following corrections with true information, and lowest following corrections with false information. We expected to show this pattern, and that recognition of corrections would decrease over time.

Our third preregistered hypothesis pertains to the role of recognizing corrections in postcorrection veracity ratings. Assessing perceived veracity and recognition of corrections using rating scales allowed us to examine within-subjects associations between those measures. This further allowed us to identify how belief updating differed based on whether corrections were recognized. Before, we showed that memory for corrections was associated with higher memory and belief accuracy (Kemp et al., 2022b, 2024a, b; Wahlheim et al., 2020). Others have also shown that belief updating is better maintained following corrections to the extent that corrections are remembered in between-subjects correlations (Swire-Thompson et al., 2023). Collectively, these findings are consistent with selective retrieval and integration accounts proposing that people will believe false details less when they can remember that those details were corrected.

Consistent with this view, our pilot results showed that recognition of corrections was associated with reduced veracity ratings. Based on these findings, we predicted that immediate reductions in the veracity ratings for false headlines would depend on whether corrections were recognized. However, we did not have an a priori hypothesis about how this dependency would change over time before we conducted Experiment 1. Experiment 1 showed that veracity ratings for false headlines increased from Phase 3 to Phase 4 based on the extent to which recognition of corrections decreased over that time, with subtle variations across formats. Consequently, we predicted a similar pattern in Experiment 2, with the provision that we were uncertain if the condition differences in such belief regression would change at the longer interval. Moreover, although we did not preregister a hypothesis about how veracity ratings and correction recognition would vary across experiments, we generally assumed, based on established associations between memory and beliefs in a similar paradigm (Swire-Thompson et al., 2023) that correction recognition would be lower at the longer retention interval in Experiment 2, and that this outcome would be associated with greater regression of veracity ratings in Phase 4.

## Methods

The present study included two experiments using identical four-phase procedures with the exception that the final phase was administered after approximately 1 week in Experiment 1 and after approximately 1 month in Experiment 2. Given the high similarity of the designs, materials, and procedures in these experiments, we report them together.

### Transparency and openness

We report how we determined sample sizes, all data exclusions, manipulations, and measures. The deidentified data upon which the study conclusions are based, the code necessary to reproduce the analyses, and the study materials are available on the OSF (https://osf.io/37vtp/). We analyzed the data using R software (Version 4.3.0; R Core Team, 2023) and the packages *car* (Version 3.1.2; Fox & Weisberg, 2019), *devtools* (Version 2.4.5; Wickham et al., 2022), *emmeans* (Version 1.8.9; Lenth, 2023), *Hmisc* (Version 5.1.1; Harrell, 2024), *lme4* (Version 1.1.34; Bates et al., 2015), *magrittr* (Version 2.0.3; Bache & Wickham, 2022); *mixedpower* (Version 0.1.0; Kumle et al., 2021); *patchwork*, version 1.1.3 (Pedersen, 2024); and *tidyverse*, version 2.0.0 (Wickham et al., 2019). We preregistered the designs and some analyses. The research complied with the Institutional Review Board at UNC Greensboro (Protocol #FY23-66).

### Participants

Participants were recruited online from Amazon Mechanical Turk using CloudResearch (www.cloudresearch.com) in all the experiments. To ensure the highest data quality, we used the CloudResearch Approved Participants feature (Hauser et al., 2022), blocked submissions with duplicated IP addresses and suspicious geocode locations, verified workers’ country and state location, and targeted MTurk workers with a HIT approval rate of 95-100%. To ensure that the headline topics—which were highly relevant to US citizens—were congruent with participants’ schemas for news content, we used the CloudResearch demographic options to target participants who indicated that the United States was their nationality and that they resided in the United States during testing. The experiments included two sessions. Participants received $4 for completing the first session, which lasted approximately 25 minutes, and $6 for completing the second session, which lasted approximately 5 minutes.

In Experiment 1, we set the sample size to match or exceed that of the pilot experiment described earlier, which included usable data from 300 participants. Because we had no basis for anticipating the attrition rate, we tested 30 participants between the end of March 2023 and the first week of April 2023 to determine how many people would return for the second session. We then launched a second wave of data collection in April 2023. Based on the attrition rate from the first wave, we made Experiment 1 available to 523 participants, to increase the likelihood of reaching a sample size of 300 or higher. We excluded data from 172 participants because 66 did not complete the first session, 22 said they randomly responded in the first session, 20 did not have any data in the first session, 18 did not return to the second session, 10 attempted the first session multiple times, nine said they randomly responded and used the internet to search for answers in the first session, eight did not have any data in the second session, five attempted the second session multiple times, five did not complete the second session, four said they randomly responded in the second session, three said they used the internet to search for answers in the first session, one said they randomly responded and used the internet to search for answers in the second session, and one said they experienced screen issues while the stimuli loaded.

The sample size in Experiment 1 was 351 participants (173 women, 177 men, and 1 non-binary) ages 20-85 years (*M* = 43.23, *SD* = 12.54). The distribution of racial identity was: 76.1% White, 11.1% Black/African-American, 4.3% Hispanic or Latino, 4% multiple races, 3.7% Asian, 0.3% American Indian or Alaska Native, 0.3% unknown, and 0.3% of participants identified as Middle Eastern or North African. The highest degree or level of school for the sample was: 40.2% four year college degree, 27.9% some college, 19.1% postgraduate or professional degree, 12.3% high school graduate, and 0.6% less than high school.

In Experiment 2, we set the sample size based on the pilot experiment and Experiment 1. We aimed to initially test 500 participants to achieve a sample size of at least 300 after attrition, but we did not have a precise basis for anticipating the attrition rate after 1 month. We tested participants from the end of August 2023 through the end of September 2023. Altogether, 570 participants started the experiment. We excluded data from 201 participants because 64 did not complete the first session, 57 did not return to the second session, 24 said they randomly responded in the first session, 20 did not complete the second session, 12 said they randomly responded in the second session, 11 attempted the first session multiple times, five said they used the internet to search for answers in the first session, three said they randomly responded and used the internet to search for answers in the first session, three did not consent in the first session, one said they randomly responded and used the internet to search for answers in the second session, and one used their mobile phone to complete the first session.

The sample size in Experiment 2 was 369 participants (206 women, 155 men, 7 non-binary, and 1 preferred not to answer) ages 20-80 years (*M* = 42.62, *SD* = 12.66). The distribution of racial identity was: 71.3% White, 11.7% Black/African-American, 7.6% multiple races, 4.9% Hispanic or Latino, 4.1% Asian, 0.3% unknown, and 0.3% of participants identified as Middle Eastern or North African. The highest degree or level of school for the sample was: 37.9% four-year college degree, 34.4% some college, 14.1% postgraduate or professional degree, and 13.6% high school graduate.

In consideration of statistical power, we conducted detailed sensitivity analyses to determine the smallest effect sizes for the coefficients of interest that could be detected with power equal to at least 80%, given our final sample sizes (see Supplementary Materials, Section 1). These were (log) odds-ratios in the case of the generalized linear mixed-effects models analyzing recognition of corrections, and standardized beta coefficients in the case of the linear mixed-effects models analyzing veracity ratings. We used *mixedpower* (Kumle et al., 2021) to conduct simulations only of models from Experiment 1, given that sensitivity was necessarily lower for Experiment 1 because the obtained sample size was smaller and the observed variance was higher than in Experiment 2. These analyses showed that we had sensitivity to detect coefficients even smaller than the observed effects for the main effects and critical two-way interactions (e.g., Headline Type × Phase) with power still equal to 80%.

### Design

**Fig. 1:**
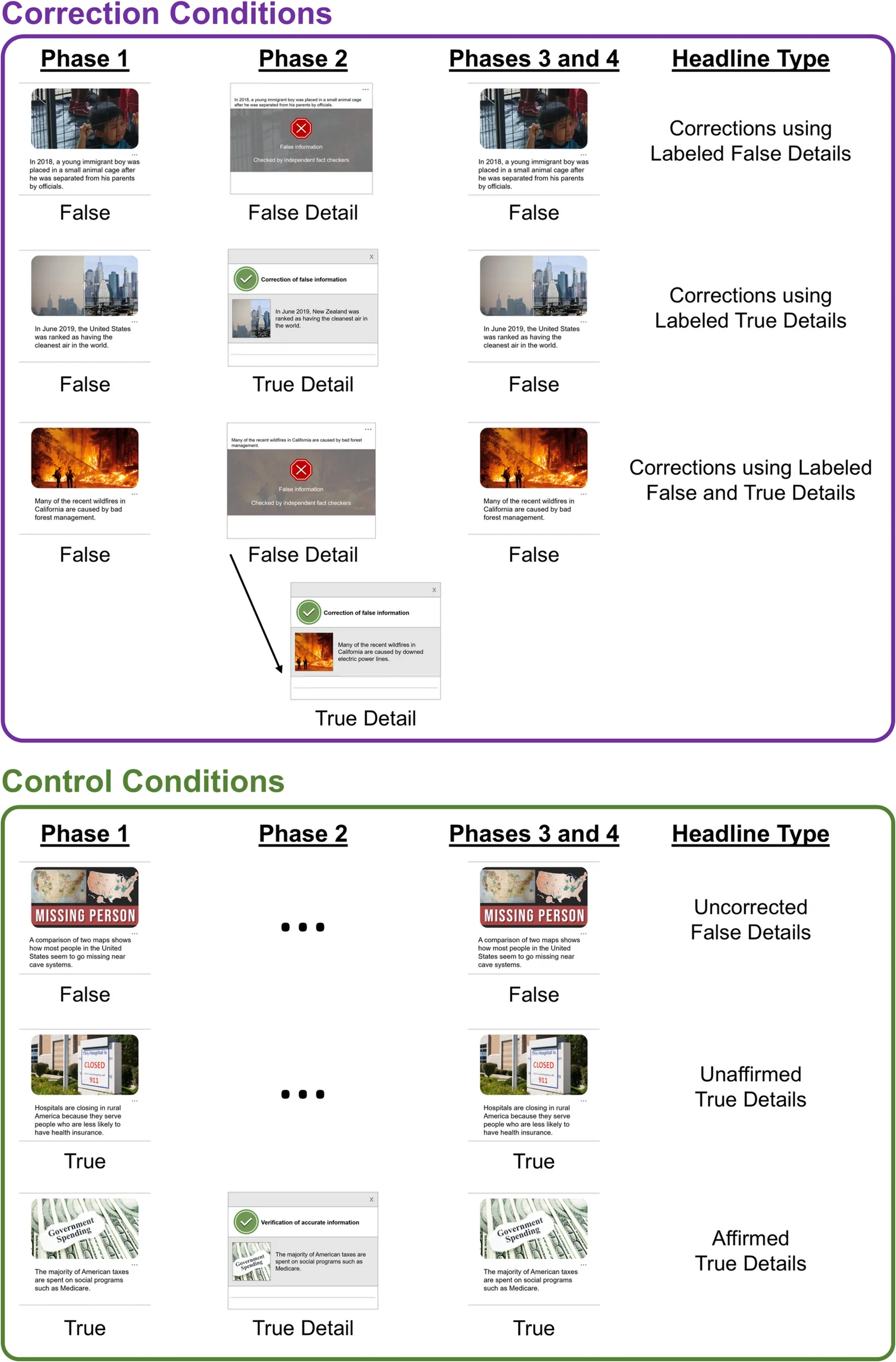
Schematic of headline type conditions. *Note.* All correction conditions (top panel, purple border) included false headlines in Phases 1, 3, and 4. In Phase 2, those conditions also included labeled false details, labeled true details, and labeled false and true details. The control conditions (bottom panel, green border) included either false or true headlines in Phases 1, 3, and 4. False headlines appeared across Phases 1, 3, and 4 without corrections in Phase 2 for uncorrected false details. True headlines appeared across Phases 1, 3, and 4 without true details in Phase 2 for unaffirmed true details, and with true details in Phase 2 for affirmed true details

The design included one within-subject independent variable, referred to as Headline Type, that included six levels. Of those six levels, three were the critical correction formats described before, and the other three were control conditions without corrections (Fig. 1).

The three correction conditions (Fig. 1, top panel) all showed unlabeled false headlines in Phase 1, labeled details in Phase 2 that corrected false headlines from Phase 1, and repetitions of the unlabeled false headlines from Phase 1 in Phases 3 and 4. The correction conditions are named based on the veracity of the details that appeared in Phase 2. Corrections using Labeled False Details only showed labeled false details in Phase 2. Correction using Labeled True Details only showed labeled true details in Phase 2. Corrections using Labeled False and True Details showed labeled false and true details, sequentially in that order, in Phase 2.

The three control conditions (Fig. 1, bottom panel) varied in terms of the veracity of the headlines that repeated across Phases 1, 3, and 4, and whether corresponding details appeared in Phase 2. Those conditions are named according to these variations. Uncorrected False Details showed unlabeled false headlines in Phases 1, 3, and 4 without corresponding details in Phase 2. Unaffirmed True Details showed unlabeled true headlines in Phases 1, 3, and 4 without corresponding details in Phase 2. Affirmed True Details showed unlabeled true headlines in Phases 1, 3, and 4 with labeled true details in Phase 2.

### Materials

**Fig. 2:**
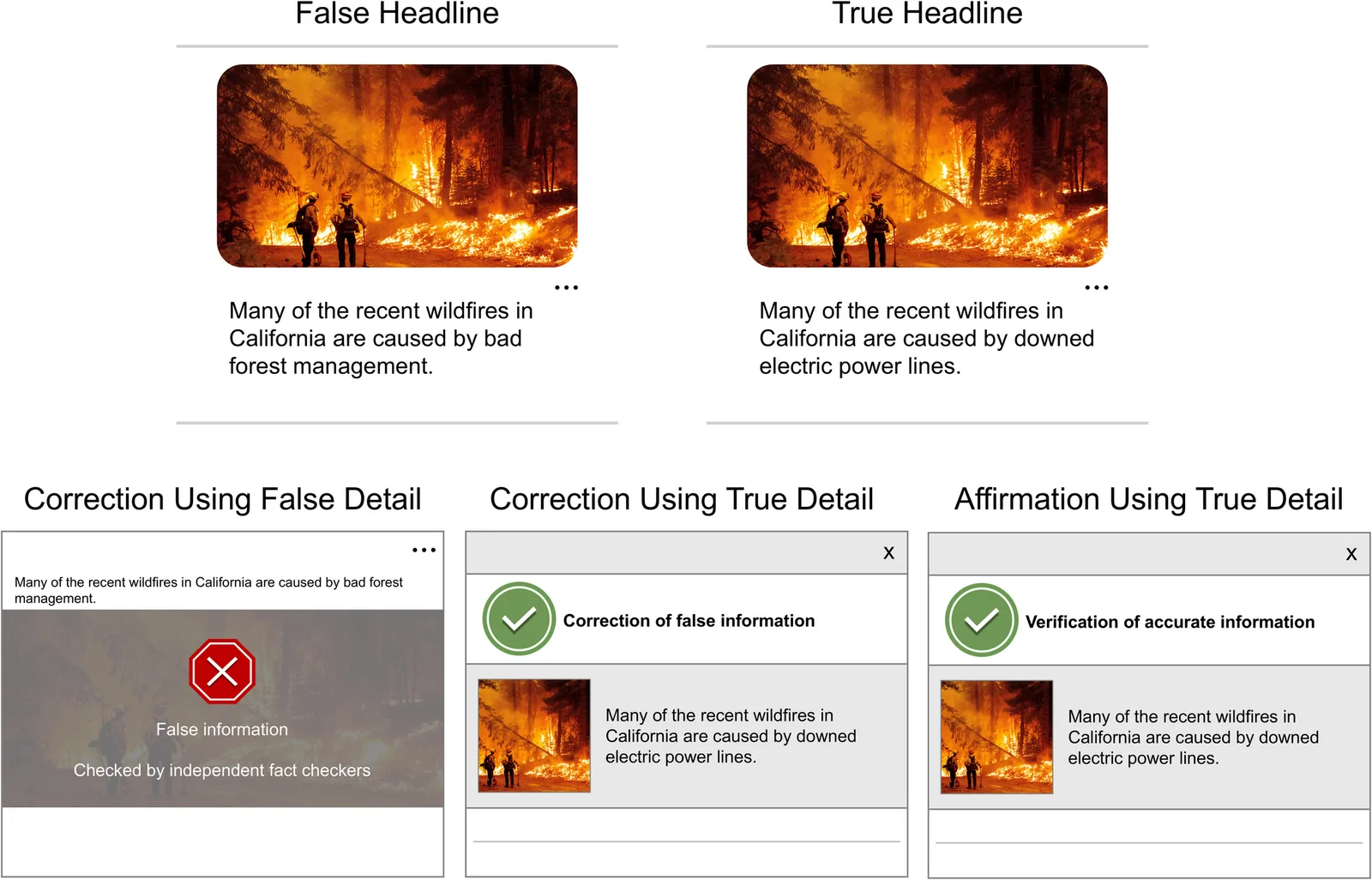
Example headline, correction, and affirmation stimuli. *Note.* Example from one headline topic depicting the false headline (top left), true headline (top right), correction repeating the false detail shown in the false headline (bottom left), correction presenting the true detail that contradicts the false detail shown in the false headline (bottom middle), and affirmation repeating the true detail shown in the true headline (bottom right)

The materials comprised 48 total headline pairs, each including one true and one false detail about the same topic. The topics came from the fact-checking websites Snopes.com, PolitiFact.com, and FactCheck.org. The stimuli encompassed a broad range of topics, such as immigration, sports, films, the environment, and health. The difference between the true and false details were either qualitative or quantitative in nature. Qualitative changes were instances where the false detail was categorically different, such as “Virginia is the No. 1 recipient of federal dollars,” compared with the true detail, “California is the No. 1 recipient of federal dollars.” Quantitative changes were instances where the false detail differed in amount. For example, the false detail could be, “The price of gasoline rose 25% in the first half of 2018,” while the true headline could be, “The price of gasoline rose 15% in the first half of 2018.” Regardless of the type of difference, each false headline was once portrayed by the media as being true before being publicly corrected. We paraphrased the original headlines so that within pairs, the text was identical except for true or false details, and designed the headline, correction, and affirmation aesthetics to resemble formats used by social media websites (for an example, see Fig. 2).

For each participant, we randomly assigned six topics to each of the correction conditions (18 total), six topics to each of control conditions that did not correct or affirm details from Phase 1 (12 total), and 18 topics to the control condition that affirmed true details in Phase 2. We assigned more topics to the last condition to equate the total true and false headlines (24 each) within Phases 1, 3, and 4 and prevent biasing veracity ratings. Note that 36 total headlines from the correction conditions appeared in Phase 2 because the headlines from the control conditions without corrections or affirmations (12 total headlines) only appeared in Phases 1, 3, and 4.

### Procedure

**Fig. 3:**
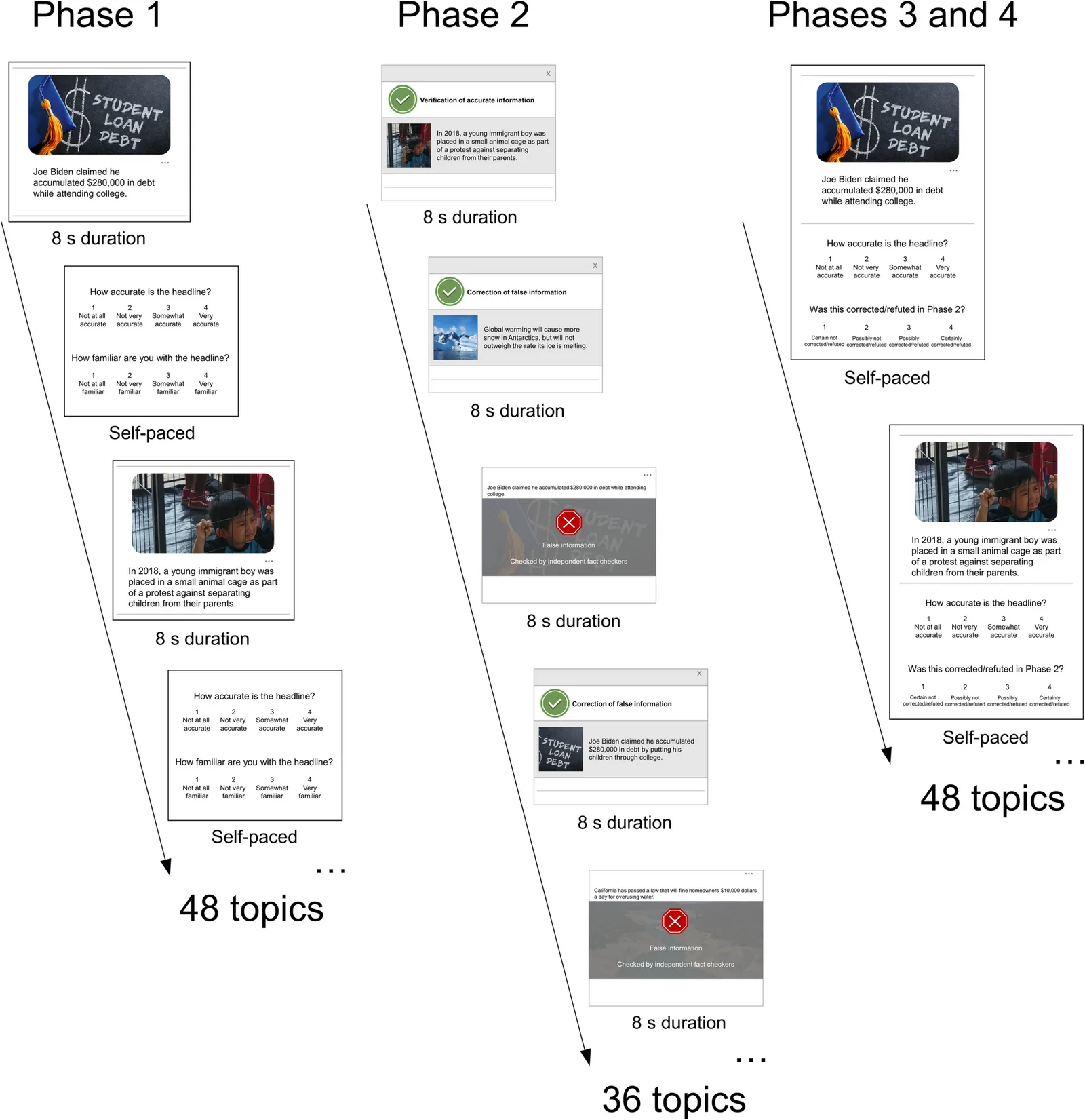
Trial structures in each phase. *Note.* In Phase 1, participants viewed each headline for 8 s, then had unlimited time to rate the accuracy and familiarity of the headline. In Phase 2, participants viewed each correction or affirmation of Phase 1 headlines for 8 s. In Phases 3 and 4, participants viewed the headlines from Phase 1 and had unlimited time to rate the accuracy of the headline and their memory for whether they were corrected in Phase 2

Qualtrics software (Provo, UT) controlled the stimulus presentation. Participants completed the task in an internet browser on their own personal computers, outside of the laboratory, and unsupervised. The experiment included two sessions and four phases. The first session included Phases 1, 2, and 3, and the second session included Phase 4. The delay between sessions was approximately 1 week (Experiment 1) or 1 month (Experiment 2). Before each phase, participants read a series of instruction slides and then answered questions about the instructions. This measure alerted participants to the critical details of the instructions before starting the task and assessed their comprehension. Most participants comprehended the instructions, as shown in the percentages of correct responses to these questions displayed in the Supplementary Materials (Section 2). We did not exclude participants based on these responses because the self-evident nature of the trials should have clarified any misunderstandings. Figure 3 displays a schematic of the trial structures in each of the phases described in what follows.

The Phase 1 instructions stated that true and false headlines would appear without information about their veracity. Participants were told that they would rate the veracity and familiarity of headlines using the full rating scales described later. Headlines appeared in the middle of the screen for 8 s each. The presentation duration was equated across headlines to prevent potential confounding between exposure time and headline type. However, as described in the Introduction, the total duration of corrections with false and true details (16 s) was twice that of the other conditions. Note that our decision to confound duration with correction format, as opposed to equating total duration between corrections with one or two claims, was based primarily on our desire to sustain participants’ attention. Our pilot work showed that around 8 s was required for participants to read all headlines completely and that shorter durations undercut comprehension for the longer headlines. That work also showed that a 16 s duration for individual headlines led to boredom and inattention, which we assumed would either provide little additional benefits to encoding or impair encoding across the task more generally.

Controlling the stimulus presentation for each headline, instead of allowing participants to freely self-pace their viewing of the posts, was necessary to properly examine how correction formats affected subsequent memory for corrections. This is a standard procedural detail in lab-based memory experiments. After 8 s, each headline disappeared. Two prompts then appeared simultaneously. Participants rated the veracity of the headline on a scale from 1 (*not at all accurate*) to 4 (*very accurate*), and their experience with the topic on a scale from 1 (*not at all familiar*) to 4 (*very familiar*). The program advanced after participants made both ratings.

The Phase 2 instructions stated that the details from headlines from Phase 1 would appear. Participants were also told that some details would affirm true details from Phase 1, others would correct false details from Phase 1, and that they should note when the Phase 2 details corrected false details. All affirmations and corrections appeared with labels indicating how the details therein related to the details from headlines from Phase 1. Images appeared in the middle of the screen for 8 s each, including for each image in corrections with false and true details that appeared sequentially. After each trial, a “Next” button replaced the image; participants clicked that button to advance. This feature was included to actively engage participants during Phase 2 because no overt judgments were required for correction stimuli.

The Phase 3 instructions stated that the true and false headlines from Phase 1 would appear in their original form. Participants were also told that their task would be to rate the veracity of each headline, as in Phase 1, and to rate the extent to which they recognize the headline being corrected in Phase 2, using the full scale for each rating. Headlines appeared above two prompts. The first prompt asked participants to rate the headline veracity on a scale from 1 (*not at all accurate*) to 4 (*very accurate*). The second prompt asked participants to rate their memory for whether the headline was corrected in Phase 2 on a scale from 1 (*certain not corrected/refuted*) to 4 (*certain corrected/refuted*). Note that we included the term “refuted” in these prompts to ensure that participants understood that we were referring to all correction types in Phase 2, including those that repeated false claims. The program advanced after participants made both ratings.

After Phase 3, participants completed questionnaires about their demographics and user experience with the program, in that order. We collected demographic information by asking participants to type their age (in years); select the gender with which they most identify (female, male, nonbinary, other, prefer not to answer); select all the ethnicities or races with which they identify (American Indian/Alaska Native, Asian, Black or African American, Hispanic or Latino, Middle Eastern or North African, Native Hawaiian or Other Pacific Islander, White, Unknown); and lastly, select the highest level of school that they completed or the highest degree they received (less than high school, high school graduate [diploma or GED certificate], some college including 2-year associate’s degree, 4-year college or university degree/bachelor’s degree [e.g., BS, BA, AB], postgraduate or professional degree including master’s, doctorate, medical, or law degree [e.g., MA, MS, PhD, MD, JD, graduate school]).

User experience questions comprised suggestions to improve the experience, problems with headline visibility, whether participants randomly responded, and whether they searched the internet for any of the news headlines. The latter three were used to exclude participants. After the questionnaires, a screen instructed participants to complete the second session a week later (Experiment 1) or a month later (Experiment 2). Participants were given grace periods for completing the second session to mitigate attrition. The average number of days between sessions was 7.09 (*SD* = 0.28, range: 6.92–9.39) in Experiment 1 and 29.98 (*SD* = 0.91, range: 27.17–31.60) in Experiment 2. During these intervals, the second author checked each data file to ensure that the participants completed all the trials in the first session on one attempt, did not randomly respond to questions, and did not search the internet to aid responses. Participants who passed these quality-control checks were placed on a list that granted them access to the second session. Eligible participants were notified up to three times about completing the second session through the CloudResearch messaging portal—once immediately after the second session became available, once 24 hours after that, and once 48 hours after the session first became available. The Phase 4 instructions were the same as in Phase 3. After completing Phase 4, participants again answered the same questions about their user experience.

### Statistical methods

We used R software (R Core Team, 2023) to conduct all analyses and examined the effects of interest using mixed-effects models from *lme4* (Bates et al., 2015). There were four sets of models corresponding to different dependent variables: familiarity, veracity ratings, and veracity ratings conditioned on correction recognition were all measured on 1–4 rating scales and were modeled with linear mixed effects (function “lmer”). Correction recognition was measured as correct/incorrect and was modeled with generalized linear mixed-effects (function “glmer,” family = binomial). All fixed effects, including Headline Type, Phase, and Correction Recognition were categorical in nature. Consequently, all contrasts were deviation-coded (e.g., −1 and +1) rather than dummy coded (e.g., 0 and 1). We followed the best practices guidance of Meteyard and Davies (2020) for selecting random effects, and the recommendations of Brauer and Curtin (2018) for addressing model convergence issues. This resulted in all the models starting with random intercepts both by-subjects and by-items, as well as random slopes by-subjects for all main effects of interest (i.e., all predictors except for any two-way or three-way interactions). We performed Wald’s χ^2^ hypothesis tests using the Anova function of the *car* package (Fox & Weisberg, 2019) and post hoc comparisons controlling for multiple comparisons using the Tukey method in the *emmeans* package (Lenth, 2021). The model specifications are available in the analysis scripts on the OSF (https://osf.io/37vtp/).

All models converged with the “bobyqa” optimizer and none had singularity warnings. The binomial models were checked for overdispersion using the *performance* package (Lüdecke et al., 2021), which confirmed no overdispersion in any model (dispersion ratios ranging 1.001–1.005, *p* values > .80). Overall model fits were assessed by R^2^ for mixed models obtained from the *MuMIn* package (Bartoń, 2022); both the marginal *R*^2^ (fixed effects alone) and conditional *R*^2^ (fixed + random effects) are reported for each model in the results section. Effect sizes for pairwise comparisons are reported as Cohen’s *d*—computed by *emmeans*, function “eff_size”—for the “lmer” models and odds ratio (*OR*) for the “glmer” models. The population estimates and 95% confidence intervals are derived from the models. The significance level was α = .05.

## Results

### Phase 1: Preexisting familiarity ratings

**Fig. 4:**
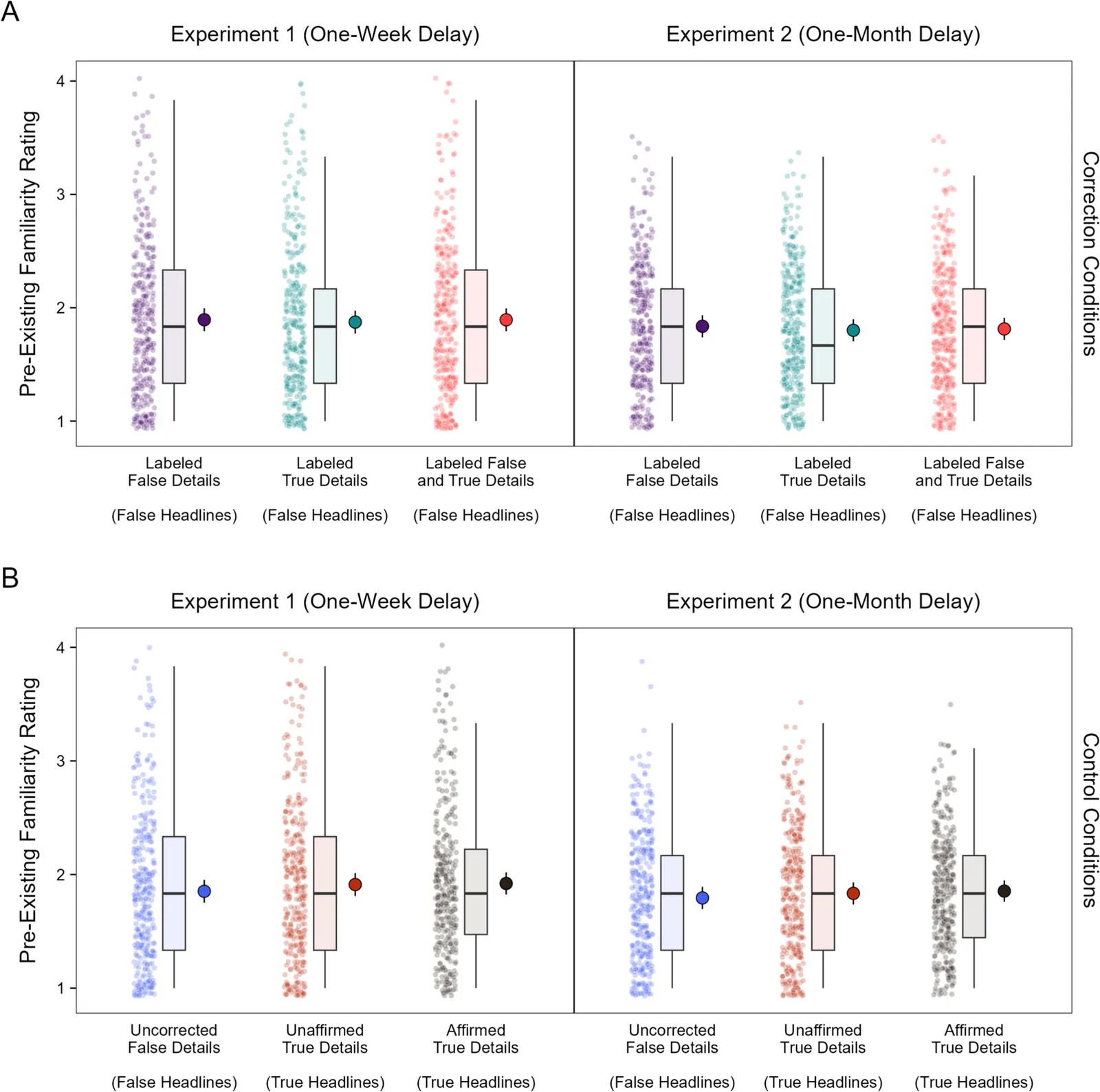
Preexisting familiarity with topics rated in Phase 1. *Note.* Familiarity ratings in Phase 1 indicating baseline experience with the headlines in the correction (**A**) and control (**B**) conditions. In each panel, the smaller leftmost points are individual participant means, box plots show interquartile ranges, medians, and the variability 1.5 times the interquartile range, and larger rightmost points are model population estimates with corresponding 95% confidence intervals (error bars)

Based on our pilot experiment, we expected that participants, on average, would be moderately unfamiliar with the true and false headlines of unclear veracity in Phase 1. However, we did not preregister this hypothesis. Figure 4 confirms that baseline familiarity was low and relatively comparable across conditions in both experiments. Pairwise comparisons revealed no significant differences across nearly all comparisons, largest *z* ratio = 2.88 and Cohen’s *d* = 0.07, *p* = .05. However, in the control conditions (panel B), ratings were significantly lower for false headlines that were not corrected (uncorrected false details) than true headlines that were affirmed (affirmed true details) in both experiments, smallest *z* ratio = 3.26 and *d* = 0.08, *p* = .01. Note that the magnitudes of these differences are small (1.85 vs. 1.92 [Experiment 1] and 1.79 vs. 1.85 [Experiment 2]). These differences may reflect that participants were more familiar with some true than false details, that the randomization of topics to conditions was imperfect, or some combination of both.

### Phases 1, 3, and 4: Veracity ratings

#### Immediate belief updating: Phases 1 to 3

**Fig. 5:**
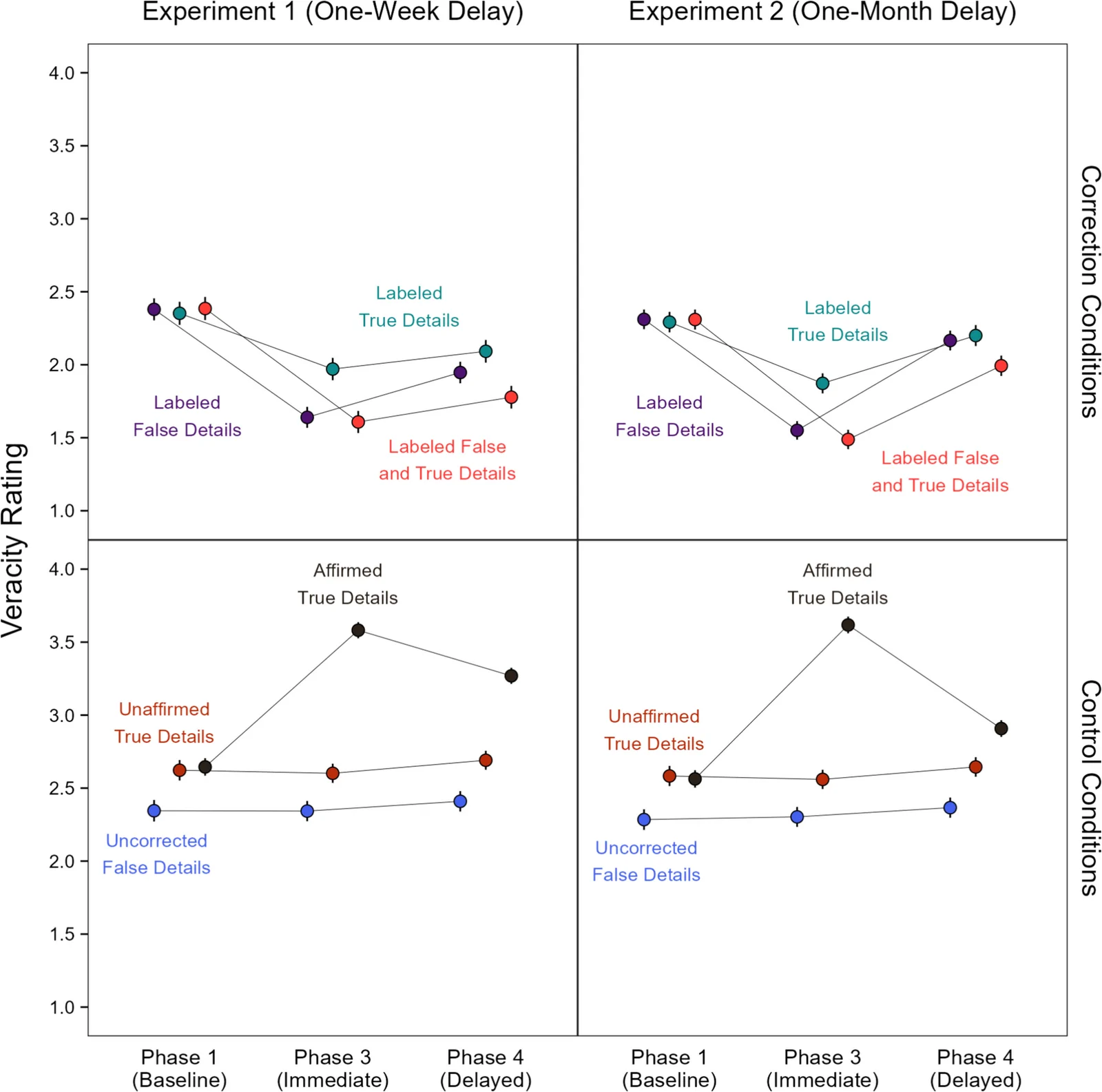
Veracity ratings: Phases 1, 3, and 4. *Note.* Veracity ratings for correction conditions (top) and control conditions (bottom). The points are model population estimates with corresponding 95% confidence intervals (error bars). Participants rated the veracity of false headlines in the three correction conditions and in the condition with uncorrected false details. Participants rated the veracity of true headlines in the conditions with affirmed and unaffirmed true details

To test our preregistered hypotheses about correction effects on beliefs in false headlines, in each experiment, we used separate models including all the Headline Type conditions across all measurement phases (Phase). The models’ marginal *R*^2^ were .278 and .267, and the conditional *R*^2^ were .456 and .389, respectively for Experiment 1 and 2. Figure 5 displays veracity ratings for false headlines in the correction conditions (top) and for false and true headlines in the control conditions (bottom). The models indicated significant effects of Headline Type, smallest χ^2^(5) = 1385.95, *p* < .001, and Phase, smallest χ^2^(2) = 52.79, *p* < .001, as well as significant interactions, smallest χ^2^(10) = 6314.03, *p* < .001. Both experiments showed comparable patterns in Phases 1 and 3.

In Phase 1, veracity ratings for false headlines did not differ across the three correction conditions and the control condition with uncorrected false details, largest *z* ratio = 1.25 and *d* = 0.05, *p* = .81. For corrected false headlines, all correction formats showed significantly lower veracity ratings in Phase 3 than Phase 1, smallest *z* ratio = 13.56 and *d* = 0.47, *p* < .001, but these reductions varied across formats as shown by differences in ratings in Phase 3. In Phase 3, veracity ratings for corrected false headlines were significantly higher following corrections with labeled true details than for corrections with labeled false details or labeled false and true details, smallest z ratio = 10.43 and *d* = 0.40, *p* < .001; ratings for false headlines did not differ between the latter two formats, largest *z* ratio = 2.42 and *d* = 0.07, *p* = .15. Moreover, in Phase 3, comparisons between the correction and control conditions showed that veracity ratings for false headlines in all correct format conditions were significantly lower than ratings for all the control conditions, smallest *z* ratio = 11.02 and *d* = 0.46, *p* < .001. These results showed that repeating false details in labeled corrections reduced perceived truth.

#### Belief regression following corrections: Phases 3 to 4

Beliefs in false headlines following corrections regressed toward baseline levels from the immediate to delayed measures, as shown by a significant increase in veracity ratings from Phase 3 to 4 for all correction conditions, smallest *z* ratio = 4.48 and *d* = 0.15, *p* < .001. However, the patterns for corrected false headlines differed between experiments in Phase 4. In Experiment 1 (left panel), veracity ratings for false headlines in Phase 4 were significantly lower for corrections with labeled false and true details than corrections with labeled false details, *z* ratio = 6.07 and *d* = 0.21, *p* < .001; ratings were also significantly lower for corrections with labeled false details than corrections with labeled true details, *z* ratio = 4.58 and *d* = 0.18, *p* < .001. Conversely, in Experiment 2 (right panel), veracity ratings were significantly lower for corrections with labeled false and true details than the other corrections formats, smallest *z* ratio = 6.74 and *d* = 0.21, *p* < .001, with the latter formats showing no significant difference, *z* ratio = 1.22 and *d* = 0.04, *p* = .83. Finally, in Phase 4 of both experiments, veracity ratings in all correction conditions were significantly lower than in the control condition with uncorrected false details, smallest *z* ratio = 5.42 and *d* = 0.20, *p* < .001. These results show that corrections with false and true details led to the most sustained reductions in false beliefs, just as on the immediate measure of belief updating in Phase 3. However, corrections with only labeled false details regressed disproportionately more over a month than a week.

#### Belief change and truth discernment in the control conditions

Although they were not the primary focus of the present study, the patterns of veracity ratings among the control conditions (Fig. 5, bottom) were noteworthy. First, repeating headlines, whether true or false, sometimes increased their perceived truth. Veracity ratings for uncorrected false details were significantly greater in Phase 4 than Phase 3 in Experiment 1, *z* ratio = 2.47 and *d* = 0.08, *p* = .04, but not in Experiment 2, *z* ratio = 2.33 and *d* = 0.08, *p* = .05. Also, veracity ratings for unaffirmed true details were significantly greater in Phase 4 than Phase 3 in both experiments, smallest *z* ratio = 3.14 and *d* = 0.10, *p* < .01. Second, the aggregate patterns showed discernment between true and false details. Veracity ratings were significantly higher for unaffirmed true details than uncorrected false details in both experiments, smallest *z* ratio = 8.38 and *d* = 0.32, *p* < .001. Third, in both experiments, affirming the veracity of true details in Phase 2 led to a significant increase from Phase 1 to Phase 3, smallest z ratio = 48.70 and *d* = 1.15, *p* < .001, which then regressed toward baseline after delays from Phase 3 to Phase 4, smallest* z* ratio = 17.63 and *d* = 0.38, *p* < .001, to a greater extent after a month than a week, similar to the regression observed in the correction conditions.

#### Individual differences in belief updating

**Fig. 6:**
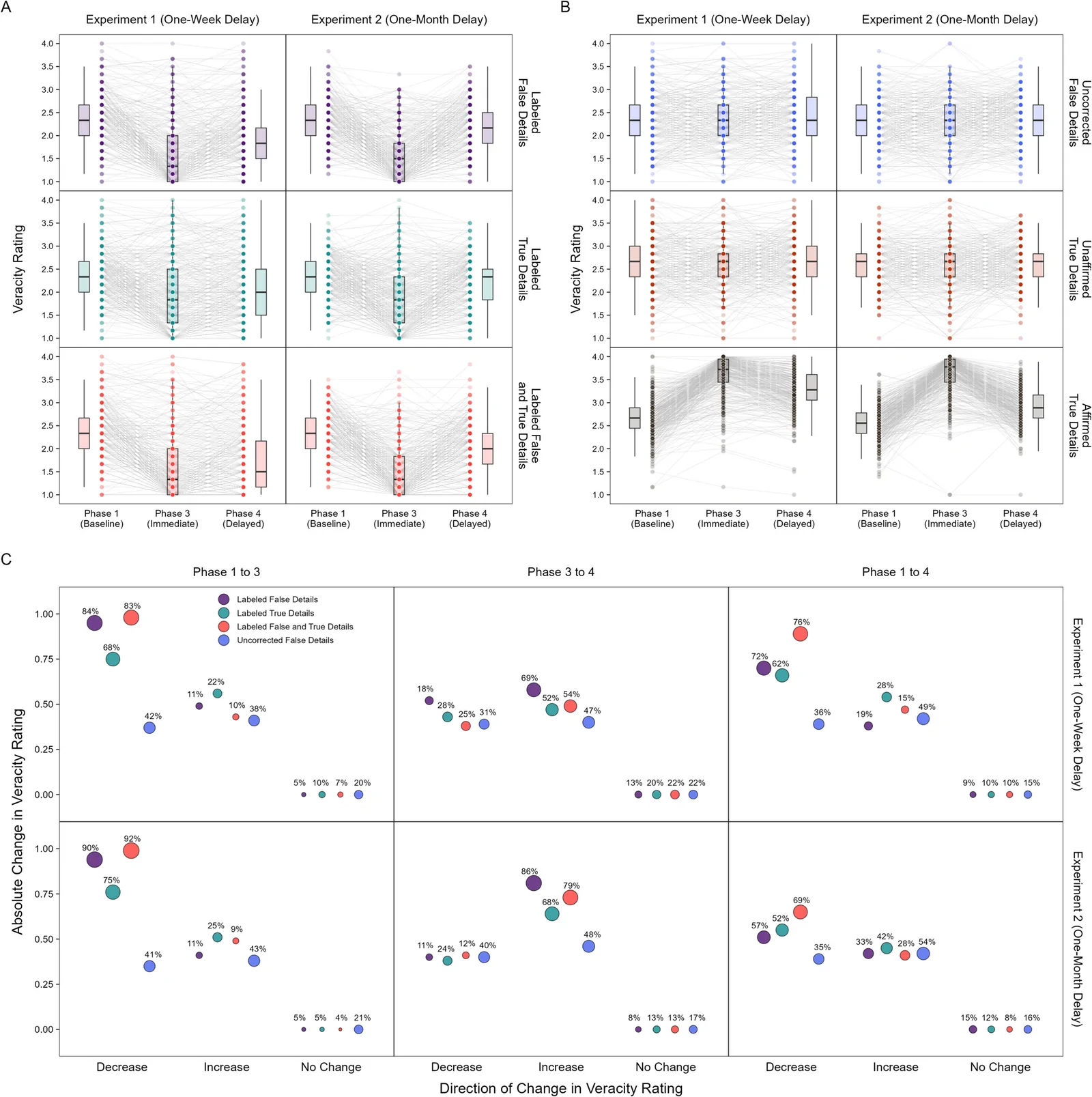
Individual differences in veracity ratings across Phases 1, 3, and 4. *Note.* Veracity ratings in Phases 1, 3, and 4 for the (**A**) correction and (**B**) control conditions. The box plots show interquartile ranges, medians, and the variability 1.5 times the interquartile range. The points are participant means. The line slopes show the magnitudes of changes in veracity ratings across phases for each participant. **C** The percentages of participants showing changes in ratings across phases in each direction and the average absolute magnitudes of those changes. The point areas indicate the differences in percentages of participants across cells

We next examined individual variability in veracity ratings across phases and conditions to provide a fuller characterization of these differences. Our goal was to determine the extent to which aggregate belief changes reflected the unique patterns shown by participants. Figure 6 displays changes in veracity ratings across phases for individual participants in the correction conditions (Panel A) and control conditions (Panel B). Figure 6C summarizes those changes by displaying the percentages of participants that showed changes in each direction or no change, as well as the average absolute magnitudes of those changes for false headlines in the correction conditions and the corresponding control condition including uncorrected false details.

Figure 6C (left) shows that the greatest aggregate reductions in veracity ratings from Phase 1 to 3, which occurred comparably for corrections with labeled false details and correction with labeled false and true details, reflected both more participants showing decreased ratings and larger magnitudes of decreased ratings compared with corrections with labeled true details. Additionally, Fig. 6C (middle) shows that the aggregate increase in veracity ratings from Phase 3 to 4 (i.e., belief regression), which varied across experiments and was consistently greatest for corrections with labeled false details, also reflected more participants showing increased ratings and greater increases in those ratings than in the other correction conditions. Finally, Fig. 6C (right) shows that the greatest aggregate sustained decrease in veracity ratings for false headlines, which was observed for corrections with labeled false and true details, also reflected both more participants showing decreased ratings and larger magnitudes of decreased ratings compared with the other conditions. More generally, although the aggregate decreases in perceived veracity of false headlines reflected most participants responding appropriately to corrections, a nontrivial percentage of participants showed changes in the direction opposite of the aggregate patterns, especially after 1 month. This suggests that despite the overall efficacy of corrections, none of the formats universally reduced the perceived truth of false headlines, which may partly reflect the extent to which participants were able to recognize corrections.

### Phases 3 and 4: Correction recognition

**Fig. 7:**
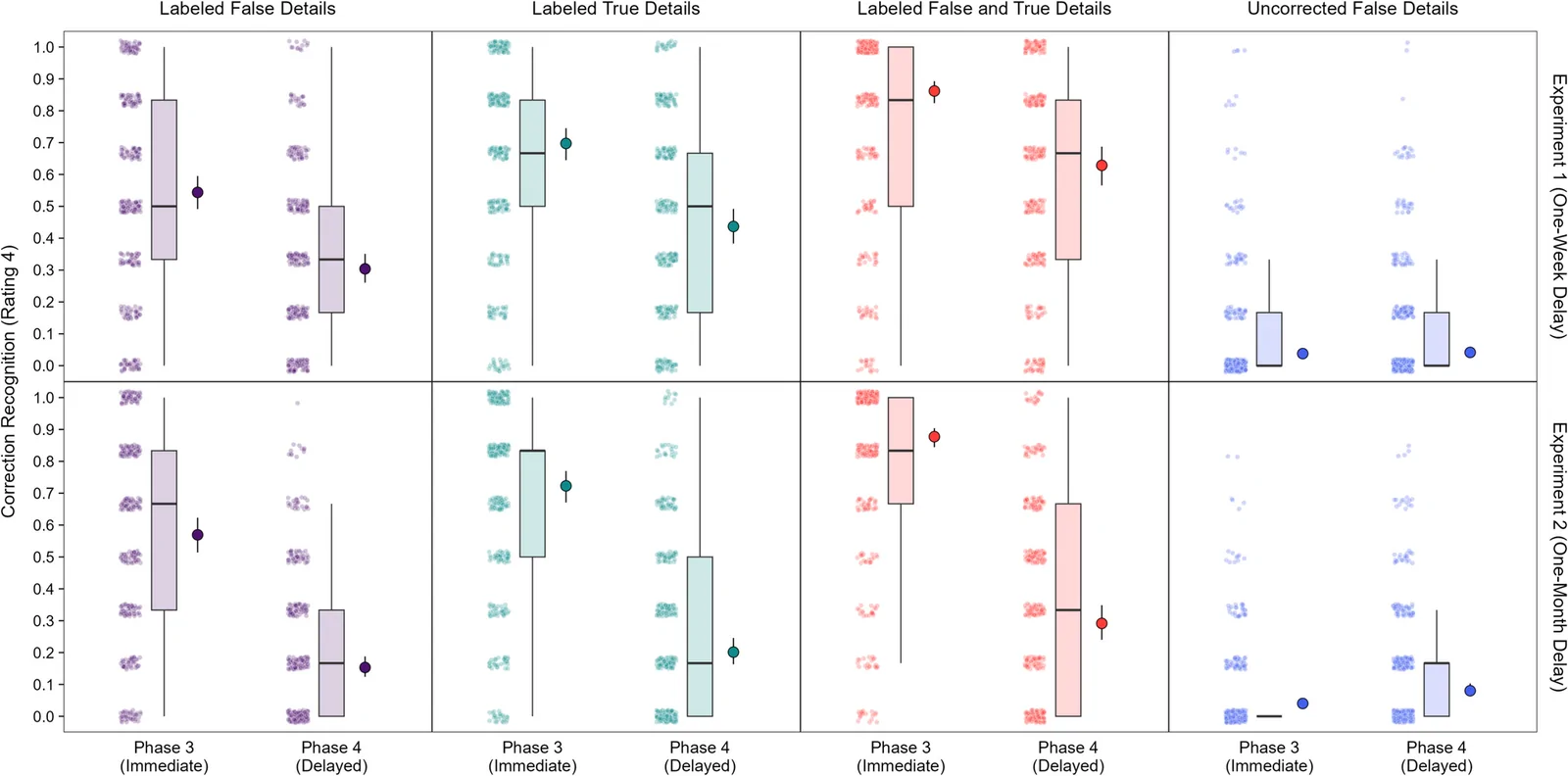
High-confidence recognition of corrections: Phases 3 and 4. *Note.* Probabilities of recognizing corrections with the highest rating (4, Certainly corrected/refuted). In each panel, the smaller leftmost points are individual participant means, box plots show interquartile ranges, medians, and the variability 1.5 times the interquartile range, and larger rightmost points are model population estimates with corresponding 95% confidence intervals (error bars). The ratings are accurate in the first three panels showing the correction conditions and inaccurate in the right panel showing the matched control condition

To test our preregistered hypotheses about the effects of correction format on memory for corrections, we used one model per experiment that included fixed effects of Headline Type, including all correction conditions and the corresponding control condition with uncorrected false details, and Phase (Phases 3 and 4). The models’ marginal *R*^2^ were .312 and .308, and the conditional *R*^2^ were .648 and .560, respectively for Experiment 1 and 2. Figure 7 displays estimates of correction recognition, which was operationally defined as the highest confidence rating that a headline was corrected (i.e., a rating of 4). As described earlier, we assumed that these mnemonic experiences were more likely to reflect recollection-based retrieval than the experiences associated with lower ratings, but we did not assume process purity. Such high-confidence recognition was accurate in the correction conditions (first three panels) and inaccurate in the control condition (right panel).

Both models indicated significant effects of Headline Type, smallest χ^2^(3) = 486.22, *p* < .001, showing significantly higher accurate correction recognition for corrections with labeled false and true details than corrections with labeled true details, smallest *z* ratio = 10.51 and *OR* = 2.11, *p* < .001, and for corrections with labeled true details than corrections with labeled false details, smallest *z* ratio = 7.12 and *OR* = 1.66, *p* < .001. Additionally, accurate correction recognition for all correction formats was significantly higher than inaccurate correction recognition for uncorrected false details, smallest *z* ratio = 18.04 and *OR* = 8.08, *p* < .001. Both models also indicated significant effects of Phase, smallest χ^2^(1) = 186.58 and *OR* = 2.28, *p* < .001, that were qualified by significant interactions with Headline Type, smallest χ^2^(3) = 84.96, *p* < .001. Accurate correction recognition was significantly higher in Phase 3 than 4 for all corrections, smallest *z* ratio = 10.74 and *OR* = 2.73, *p* < .001, whereas inaccurate correction recognition for uncorrected false details was not significantly different between phases in Experiment 1, *z* ratio = 0.70 and *OR* = 1.10, *p* = .48, and significantly higher in Phase 4 than 3 in Experiment 2, *z* ratio = 4.81 and *OR* = 2.08, *p* < .001. Overall, participants’ ability to distinguish between corrected and uncorrected false headlines diminished over time, especially at the longer 1-month delay.

### Phases 1, 3, and 4: Veracity ratings conditioned on correction recognition

Next, we tested our preregistered hypothesis about how correction effects on veracity ratings should differ based on correction recognition. One purpose of the following analyses was to examine whether sustained decreases in veracity ratings for false headlines depended on whether participants recognized corrections. We examined this by comparing veracity ratings for false headlines when participants recognized corrections with the highest confidence (Recognized) or did not recognize corrections with the highest confidence (Not Recognized). Another purpose of the following analyses was to examine the degree to which not recognizing corrections was associated with increased veracity ratings for false headlines, and if ratings would backfire by rising beyond baseline levels in Phase 1. We did this by comparing baseline ratings for corrections (Phase 1) with ratings after corrections, after longer delays (Phase 4).

**Fig. 8:**
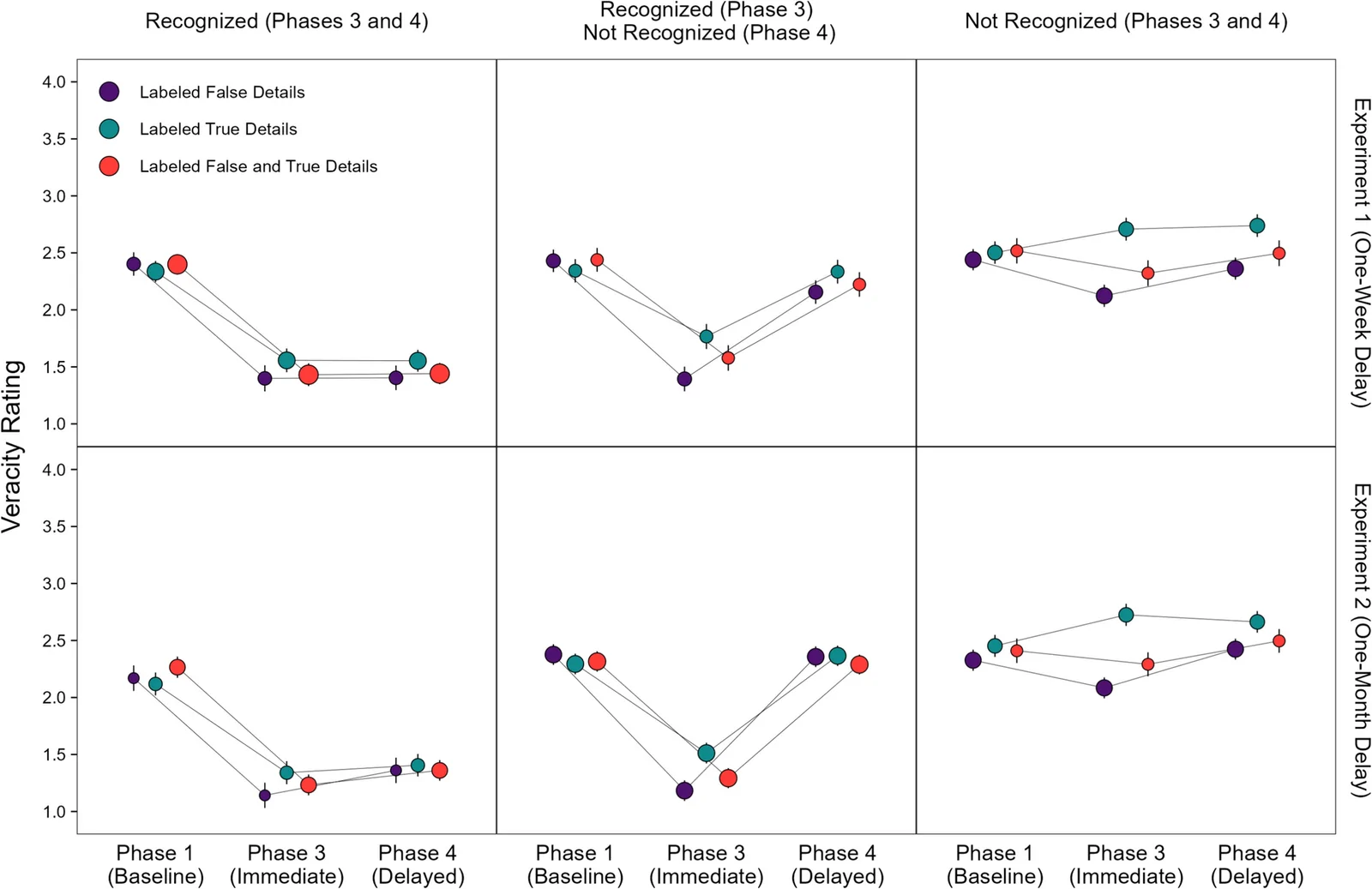
Veracity ratings in the correction conditions (Phases 1, 3, and 4) conditionalized on combinations of high-confidence recognition of corrections in Phases 3 and 4. *Note.* Veracity ratings for correction conditions when corrections were recognized in Phases 3 and 4 (left), recognized in Phase 3 but not in Phase 4 (middle), and not recognized in either phase (right). The points are model population estimates with corresponding 95% confidence intervals (error bars). The point areas indicate the differences in observations across conditional cells

We made these comparisons using separate models for each experiment. The models included the following fixed effects: Headline Type (including the three correction conditions), Phase (including Phases 1, 3, and 4), and Correction Recognition (including three of the four possible combinations across Phases 3 and 4). For Correction Recognition, we conditionalized veracity ratings on whether corrections were recognized in both Phases 3 and 4, recognized in Phase 3 but not recognized in Phase 4, or were not recognized in Phases 3 and 4. We did not analyze instances when corrections were not recognized in Phase 3 but were recognized in Phase 4: The processes underlying these instances were difficult to infer because correction recognition responses in Phase 4 may often reflect guessing instead of legitimate memories of corrections that were inaccessible in Phase 3. Table 1 shows that all effects were significant except the three-way interaction in Experiment 2. The models’ marginal *R*^2^ were .192 and .255, and the conditional *R*^2^ were .500 and .393, respectively for Experiment 1 and 2. Figure 8 displays the conditional veracity ratings.

**Table 1:** Model results for veracity ratings conditionalized on correction recognition

	Experiment 1			Experiment 2		
Effect	χ^2^	*df*	*p*	χ^2^	*df*	*p*
Headline Type	90.52	2	<.001	101.07	2	<.001
Phase	727. 06	2	<.001	1,272.07	2	<.001
Recollection	380.66	2	<.001	1,864.10	2	<.001
Headline Type × Phase	152.58	4	<.001	172.19	4	<.001
Headline Type × Correction Recognition	47.01	4	<.001	74.11	4	<.001
Phase × Correction Recognition	1,096.97	4	<.001	1,567.94	4	<.001
Headline Type × Phase × Correction Recognition	16.50	8	= .036	13.27	8	= .103

Belief changes depended on when participants recognized corrections, as indicated by significant Phase × Correction Recognition interactions. When corrections were recognized in Phases 3 and 4 (left panel), veracity ratings for false headlines were significantly lower in those phases than in Phase 1, smallest* z* ratio = 25.64 and *d* = 1.04, *p* < .001. When corrections were recognized in Phase 3 but not Phase 4 (middle panel), veracity ratings for false headlines were significantly lower in Phase 3 than 1, smallest *z* ratio = 23.99 and *d* = 1.11, *p* < .001, and significantly higher in Phase 4 than 3, smallest *z* ratio = 21.23 and *d* = 0.88, *p* < .001. Comparisons between Phases 1 and 4 showed that, in Experiment 1, veracity ratings for false headlines were significantly lower in Phase 4 than 1 for corrections with labeled false details and with labeled false and true details in Experiment 1, smallest *z* ratio = 4.01 and *d* = 0.29, *p* < .001, whereas ratings did not differ between those phases for corrections with labeled true details, *z* ratio = 0.15 and *d* = 0.01, *p* = .99; in Experiment 2, veracity ratings were not significantly different between Phases 1 and 4, largest *z* ratio = 1.84 and *d* = 0.09, *p* = .16.

When corrections were not recognized in Phases 3 and 4 (right panel), veracity ratings for false headlines that were corrected with labeled true details were significantly higher in Phases 3 and 4 than Phase 1, smallest *z* ratio = 4.37 and *d* = 0.28, *p* < .001, and were not significantly different between Phases 3 and 4, largest *z* ratio = 1.40 and *d* = 0.08, *p* = .34. Conversely, for corrections with labeled false details and with labeled false and true details in Experiment 1, veracity ratings were significantly lower in Phase 3 than 1, smallest* z* ratio = 3.39 and *d* = 0.26, *p* < .01, significantly higher in Phase 4 than 3, smallest *z* ratio = 3.17 and *d* = 0.16, *p* < .01, and not significantly different in Phases 1 and 4, largest *z* ratio = 1.85 and *d* = 0.11, *p* = .15. In Experiment 2, veracity ratings for false headlines were significantly lower in Phase 3 than 1 for corrections with labeled false details, *z* ratio = 5.70 and *d* = 0.31, *p* < .001, and not significantly different between those phases for corrections with labeled false and true details, *z* ratio = 2.06 and *d* = 0.15, *p* = .10. Veracity ratings for false headlines corrected with labeled false details were significantly higher in Phase 4 than 1, *z* ratio = 2.39 and *d* = 0.31, *p* < .05, and not significantly different between those phases for corrections with labeled false and true details, *z* ratio = 1.55 and *d* = 0.15, *p* = .27. Taken together, these ratings show that recognizing corrections was associated with reductions in the perceived veracity of false headlines. Conversely, failing to recognize corrections was associated with belief regression and, sometimes, familiarity backfire. These findings highlight that the corrections improve belief accuracy to the extent that they promote high quality memories including contextual details, such as veracity information.

### Summary

The comparisons of veracity ratings for false headlines across experiments showed that the immediate improvement in belief accuracy after corrections diminished over time. Such belief regression was stymied when participants recognized corrections, and corrections with labeled false and true details led to the best subsequent recognition of corrections. Together, these findings suggest that mnemonic processes played a role in the advantage of corrections that included false and true details. Interestingly, when corrections were not recognized, false beliefs were *greater* than at baseline in some instances where corrections used only false or only true details. Collectively, these results suggest that although corrections are generally effective, such efficacy depends on the extent to which memories provide a valid basis for perceptions of veracity.

## General discussion

The present study revealed a mnemonic mechanism underlying differences in the efficacy of corrections of false headlines for improving belief accuracy. The main theoretical insight is that features of corrections that promote encoding and subsequent recognition that headlines were corrected may also lead to more accurate perceptions of truth and falsity over the long term. Supporting this view, the format that promoted the best recognition of corrections (i.e., corrections with labeled false and true details) yielded the lowest veracity ratings for false headlines after 1 week and month. These long-term benefits are reminiscent of work showing short-term benefits of reminder-based corrections on the accuracy of beliefs in immediately recalled headline details (Kemp et al., 2022b; Wahlheim et al., 2020). The present study also showed that correction effects varied across participants. The aggregate benefits of using false and true details during corrections reflected more participants reporting larger reductions in veracity rating for false headlines. However, many participants showed either no changes or even increased veracity ratings. These differences likely reflected variations in recognition of corrections. Supporting this assertion, within-subjects analyses showed that when participants did not recognize corrections, they showed little (if any) reduction in veracity ratings for false headlines. Collectively, the insights about mnemonic processing gained here could inspire correction methods that promote associative encoding of false and true details.

The present findings have implications for leading views of the effects of repeating false details during corrections on beliefs. Accounts of repetition-induced truth predict that corrections that repeat false information will increase veracity ratings for false headlines to the extent that contextual details, including information veracity provided by labels, cannot be recollected (for a review, see Unkelbach et al., 2019). This led to the prediction that corrections repeating false details, with or without true details, should be less effective at reducing beliefs in false headlines than corrections with only true details on delayed measures when participants are less likely to recollect contextual details. Additionally, the familiarity backfire account (Schwarz et al., 2007; Skurnik et al., 2005) led to the extreme prediction that after delays that undermine recollection corrections repeating false information could increase beliefs in false headlines above baseline. However, contrary to these predictions, all the correction formats here produced aggregate reductions in veracity ratings from the first to final phase, and such reductions were greater for corrections using false and true details than corrections that only repeated false details or only showed true details. Moreover, final aggregate veracity ratings for false headlines were never greater after corrections using only false rather than only true details.

The patterns of veracity ratings here were more compatible with integration accounts proposing that formats that promote the co-activation of false and true information will best support memory for headline veracity. Specifically, the consistent finding that correction using false and true details led to the most sustained reduction in veracity ratings for false headlines suggests that those details were encoded together best when they appeared in close temporal proximity. Moreover, the measure of correction recognition showed that this correction format best promoted memory for corrections, which likely indicated better recollection of veracity information over time. Overall, these findings are compatible with the KReC framework (Kendeou et al., 2014, 2019) of belief updating, which proposes that true information becomes integrated with false information, and that network activation shifts continually more towards true than false information. However, although this account is supported by findings from paradigms using refutational texts and in general by the advantage of reminder-based corrections reported here and elsewhere, it does not fit perfectly with a key aspect of the present findings. Namely, the results showing that reductions in veracity ratings for false headlines diminish over time is incompatible with the proposal that activation shifts from false to true information. This limitation of the KReC may reflect that it was proposed to understand knowledge comprehension processes with less emphasis on the role of episodic memories per se in belief updating.

The relationship between beliefs and memory in misinformation correction paradigms like those used here may be explained by the MFC framework (Jacoby et al., 2015; Wahlheim & Jacoby, 2013). The MFC proposes that co-activation of conflicting information during encoding can promote subsequent memory but can also lead to interference. The key mechanism is whether recollection-based retrieval can be engaged. Here, this can explain the advantages of corrections with false and true information for reducing false beliefs as reflecting better encoding and recollection of those details. However, the account was not originally proposed to explain belief updating. If the account is modified to assume that recollected memories are a valid basis for veracity ratings, then it can explain the bulk of the present findings, including that correction effects were best sustained when participants recognized corrections. However, this modified account cannot completely explain the present findings because corrections using only true details led to the least adaptive belief updating, but not to the worst correction recognition. This may have reflected participants remembering correction displays with true details reasonably well but mistaking some for affirmations because of the feature overlap in display formats (see Fig. 2).

Although aspects of the present findings are compatible with both integrative encoding accounts, we did not design the present experiments to adjudicate between those accounts. Our goal was to establish whether integrative encoding accounts are more useful than accounts of repetition-induced truth for predicting correction format effects using naturalistic stimuli. To more precisely adjudicate between the KReC and MFC accounts, future studies could use variants of the current paradigm that are more similar to lab-based memory experiments and therefore less ecologically valid. This increase in experimental control would enable direct manipulations of opportunities to engage integrative encoding. For example, one could vary the extent to which corrections fill knowledge gaps by manipulating semantic properties of stimuli. Additionally, one could directly measure retrieval of false information during corrections to identify the role of existing memory reactivation, its co-activation with new information, and the downstream consequences for memory accuracy upon which beliefs are based over time, similar to earlier studies (Kemp et al., 2022a, 2024b). Either approach should lend to techniques that identify differences in encoding of misinformation and corrections based on patterns of neural activation, as studied before (Brydges et al., 2020; Guo et al., 2025).

Moreover, the role of correction recognition, which is central to the MFC account, but not to the KReC account, could be validated using converging measures of recollection-based retrieval. As described in the Introduction, we assumed based on the extant source memory literature that the correction recognition measure leveraged here captured recollection-based retrieval, albeit imperfectly because of the process impurity of confidence measures. Future studies could use other subjective reports, such as modified versions of the remember/know procedure (Tulving, 1985), that ask participants directly about their recollection of correction experiences. To more objectively assess the role of recollection, studies could also use process dissociation procedures (Yonelinas & Jacoby, 2012) that require recollection of veracity information to endorse true information and reject false information, while opposing false information familiarity that is unaccompanied by memory for veracity information. Process dissociation procedures could potentially be combined with contemporary modeling approaches that estimate the contributions of recollection types and veracity perceptions, similar to work on the mnemonic bases of truth and falsity judgments (e.g., Niedziałkowska & Nieznański, 2021).

When considering the ability of integration accounts to explain the present findings, one must also consider a methodological limitation raised earlier. To balance experimental control with ecological validity, the correction conditions varied along two dimensions—the veracity of the details and the presentation duration. Corrections using only false details were designed to simulate instances when social media users saw flagged misinformation but did not click through to see corrective details, whereas corrections using false and true details simulated instances when corrective details were also viewed. The latter instances presumably created opportunities for co-activation and integration, but they also created longer study episodes. Participants may have sometimes used remembered durations as one basis for veracity ratings and correction recognition. Although we cannot disentangle the extent to which recollection of integrated representations and memory for duration contributed to veracity judgments after corrections, the compatibility of the present findings with prior research suggests that integration, at the very least, played a role. Specifically, studies using episodic memory tasks with stimuli content akin to those used here consistently showed that corrections using false and true details strongly promoted subsequent recall of true details (Kemp et al., 2022b; Wahlheim et al., 2020). This result cannot be explained by a duration account because the total exposure time was longer for false than true details, which could lead to proactive interference. However, true details were recalled better when false information appeared in reminder-based corrections than when corrections did not repeat false information. Collectively, these findings suggest that integrative encoding promoted recollection-based retrieval here, but future studies using more stringent control conditions that remove study duration differences are needed to verify this.

Future studies matching the study duration between corrections presenting either one or two pieces of information would illuminate the roles of encoding time and encoding quality in the observed benefits of corrections with false and true information to subsequent correction recognition. As described in the Introduction, we did not present corrections with only true or false information for 16 s each because our pilot work showed that participants required far less time to comprehend the posts and they became inattentive due to boredom. If this time-matching approach were used in a subsequent experiment to resolve this lingering issue, we expect that corrections with only one piece of information could be slightly better recognition in aggregate because some headlines may have still benefitted from extra study time. However, such recognition would be unlikely to rise to the level observed for corrections with false and true information due to the qualitatively superior encoding engendered by integration. Moreover, excessive study time could create the unwanted consequence of more attentional lapses, thus globally impairing correction recognition. To mitigate potential lapses, one could shorten the study duration for each post within corrections with false and true information, but this could prematurely interrupt ongoing encoding, thus creating another confound. If a perfect solution could be reached that would allow matched encoding time and complete but not excessive encoding, and corrections with false and true information still reigned supreme, then one could infer that such benefits reflected various cognitive processes, such as upregulated attention during the change from false to true information and integrative encoding of that information. However, that approach would still have the limitation of being unable to isolate mechanisms.

This limitation notwithstanding, the present findings are valuable in showing a role for correction recognition in correction format effects on the reduction of beliefs in false headlines using a paradigm with veracity ratings for all headlines. This extends on studies focusing on memory updating because those studies assessed the perceived veracity of false details for only the subsets of responses when recollection was most likely to have failed (i.e., intrusion errors). This memory-first approach estimated belief accuracy less precisely by not measuring the perceived veracity of false details from headlines for which participants could also remember true details. Such misestimation was avoided in a study examining the association between belief updating and memory for corrections in which participants rated their beliefs for all items (Swire-Thompson et al., 2023). Between-subjects comparisons showed that on average corrections reduced the perceived veracity of false statements to the extent that participants remembered corrections. Despite the value of showing associations between beliefs and memory, that study did not examine the role of recognizing corrections with high confidence in the efficacy of various correction formats. The present study extended that work by showing that corrections using false and true details led to the most sustained reduction in veracity rating for false headlines and the best recognition of corrections. The within-subjects associations between correction recognition and veracity ratings shown here also provide more specificity in the degree to which false beliefs regress, remain stable, or backfire over time.

These theoretical advances motivate research aimed at understanding the mechanisms of optimal correction formats. One goal here was to use a naturalistic approach to identify differences in correction efficacy that are likely to generalize. We did this by presenting content from fact-checking websites in formats like those on social media sites to a US sample that is more diverse than a typical university department subject pool. This approach follows work examining correction durability for widespread misperceptions. For example, repeated corrections including false details about COVID-19 led to sustained reductions in false beliefs, but this improvement in belief accuracy disappeared at longer intervals when perceived truth was assessed for only the false content (Carey et al., 2022). While these findings show that correction effects are transient, the contributions of misperceptions for specific content to aggregate belief changes, such as those for which corrections could not be remembered, were unclear. Conversely, the present results suggest that corrections using false headlines can sustain updated beliefs when correction formats promote effective encoding.

Identifying variables that lead to individual differences in correction efficacy is also paramount for understanding how correction differences arise in aggregate. The present study showed patterns of aggregate belief changes that were reflected in individual participants, but the consistency and magnitude of changes varied. Although this study was not designed to illuminate the contribution of other variables to the key outcomes, some possibilities may be tested. Belief updating differences may reflect the ability to associatively encode false and true details. If so, people with higher working memory capacity may benefit more from corrections with false and true details, as people with lower working memory can be more susceptible to corrected misinformation (Brydges et al., 2018; but see Sanderson et al., 2021). Relatedly, attention is necessary for noticing changes. Therefore, people whose attention lapses more frequently may benefit less from corrections, as studies have shown that people who sustain their attention less well when studying changes show poorer memory updating (Garlitch & Wahlheim, 2020; Wahlheim et al., 2025). Finally, the self-relevance of topics may determine correction efficacy, with entrenched misconceptions being difficult to correct (for a review, see Flynn et al., 2017).

The present study had limitations other than those noted before. First, we designed the experiments to optimize the sensitivity to detect correction effects on subsequent beliefs and memory, and to preclude decision biases created by imbalanced proportions of true and false details. We did this by including more headlines than consumers would typically read on social media in one sitting and a higher ratio of false to true headlines than what is commonly viewed on news feeds. These features created functionally longer lists and less distinctive corrections that could have produced lower memory for headlines and their veracity than what would occur in daily life. Second, we avoided using headline topics and frames that were strongly politicized to minimize interactions with participant worldviews. However, social media headlines do not always appear in sterile formats. It is therefore unclear whether the present correction effects would generalize to political topics across consumers with diverse views. Third, by conducting this research on the internet, we were able to test samples that were more diverse than typical university undergraduate participant pools. However, there was still a prominent concentration of highly educated, younger and middle-age, white adults from the US, which limited the generalizability of these findings to other backgrounds and cultures. Fourth, the present study did not assess the extent to which participants accepted corrections or instead preferred to believe false headlines, nor did it assess whether corrections were remembered better when the topics were intrinsically interesting. We assumed that by randomly assigning topics to conditions, we minimized any contributions of these variables to differences created by manipulating correction conditions. Future studies could identify the roles of these potential moderators by modeling the contribution of participants’ acceptance of corrections and intrinsic interest in headline topics.

A final limitation concerns the treatment of data quality in our samples. We preregistered exclusion criteria that reflected standard practices at the time of data collection, relying on self-reports of engagement. However, we acknowledge that behavioral indicators, such as reaction time variability, could have served as converging indices of attentional engagement. Because we did not preregister such measures, we chose not to apply post hoc exclusions based on them to avoid introducing experimenter degrees of freedom. Future studies could increase the robustness of findings by incorporating more stringent data quality protocols, including preregistered and empirically validated behavioral indicators, as well as safeguards against the use of artificial intelligence-assisted responding. Despite this limitation, we note that the patterns of results across experiments were orderly and consistent with existing theories and prior empirical findings.

## Conclusion

The present study showed that corrections using false and true headlines produced the most durable reductions in false beliefs in news headline content from the internet. Critically, the advantage of this correction format strongly reflected the contribution of improved recognition of corrections. Showing these findings in a belief updating paradigm provides the theoretical contribution that a dual-process perspective that incorporates assumptions from integrative encoding and repetition-induced truth accounts reasonably accounts for these findings by describing how correction formats produced memory differences that were substantially, but not perfectly, associated with belief updating. These findings extend work showing the superiority of corrections using false and true details with immediate tests (e.g., Ecker et al., 2017; Kemp et al., 2022b; Wahlheim et al., 2020) and associations between forgetting and belief regression (Swire-Thompson et al., 2023). The naturalistic approach of using false headlines and fact-checked corrections in diverse US samples may inform everyday interventions. Considering individual differences that influence correction efficacy, such as cognitive abilities and world-views, may improve our understanding of the role of memory in false belief reduction and improve the selection of everyday techniques for correcting false information on social media.

## Supplementary Information

Below is the link to the electronic supplementary material.

Supplementary file1 (DOCX 705 KB)

## Data Availability

The datasets generated during and analyzed during the current study are available in the Open Science Framework repository (https://osf.io/37vtp/).

## Code availability

The analysis code scripts for this study are available in the Open Science Framework repository (https://osf.io/37vtp/).

## Declarations

### Author note

National Science Foundation Grant Award # 2224565 supported this research. We disseminated some of the ideas and data reported here in a talk at the 64^th^ Annual Meeting of the Psychonomic Society in San Francisco, CA. The materials, data, and analysis scripts are available on the Open Science Framework (https://osf.io/37vtp/).
