# Supplementary material for: Sustaining corrected beliefs in false news headlines over time: The roles of correction format and recognizing corrections
Source: Mem Cognit. 2025 Aug 6;54(7):2513–37. doi: 10.3758/s13421-025-01760-7 (PMC13751715; doi:10.3758/s13421-025-01760-7)
Supplement: Supplementary file 1 [file 13421_2025_1760_MOESM1_ESM.docx]

**Supplementary Materials**

1. **Sensitivity** **Analyses**

Sensitivity analyses determined the smallest effect size of interest that could be detected with power = 80%, given the sample sizes and variance empirically obtained in the experiments. We used the R package *mixedpower* (version 0.1.0; Kumle et al., 2021), which uses a model simulation approach, to estimate the statistical power for a range of effect sizes (beta coefficients from the mixed models) for each of the predictors of interest: Headline Type, Phase, Correction Recognition, and their interaction terms. We did this for each of the three mixed-effects models: Veracity Ratings, Correction Recognition, and Veracity Ratings Conditionalized on Correction Recognition, based on the data from Experiment 1^[1]^. We conducted 1,000 simulations for each model (200 per effect size).

Below, the results are summarized in Tables S1-S3 and displayed in Figures S1-S3. The effect sizes are presented here as unstandardized beta coefficients for the models with Veracity Ratings as the dependent measure (both unconditional and conditionalized on correction recognition), and standardized log odds ratios for the models with Correction Recognition as the dependent measure. The tables summarize the results in terms of (a) the smallest beta coefficients that could be detected with 80% power, and (b) the range of empirically observed beta coefficients, to provide a point of comparison. The figures depict the increase in power (y-axis) as the effect size increases (x-axis), with the dashed red line indicating the point at which 80% power is reached. For example, in Experiment 1, when regressing Headline Type on Veracity Ratings, the coefficients for the contrasts of corrections using labeled false details compared to corrections using labeled true details, corrections using labeled false details compared to correction using labeled true and false details, were 0.150 and 0.065, respectively, and in Experiment 2, the same coefficients were 0.113 and 0.079 (Table S1). We had sensitivity to detect coefficients as small as ≈0.045 with power still equal to 80% (Figure S1, top left).

**Table S1**

Summary of Sensitivity Analyses for Veracity Ratings

| Effect | Sensitivity |  | Experiment 1 |  | Experiment 2 |
| --- | --- | --- | --- | --- | --- |
|  |  |  |  |  |  |
|  |  |  |  |  |  |
| Headline Type | 0.046 |  | *0.065, 0.150* |  | *0.079, 0.113* |
|  |  |  |  |  |  |
| Phase | 0.029 |  | *0.091, 0.164* |  | *0.011, 0.159* |
|  |  |  |  |  |  |
| Headline Type × Phase | 0.115 |  | *0.038, 0.358* |  | *0.053, 0.341* |
|  |  |  |  |  |  |

*Note.* The column Sensitivity reports the smallest beta coefficients that could be detected with 80% for each of the effects of interest. The columns Experiment 1 and Experiment 2 report the range of coefficients empirically observed as (*minimum, maximum)*.

**Table S2**

Summary of Sensitivity Analyses for Correction Recognition

| Effect | Sensitivity |  | Experiment 1 |  | Experiment 2 |
| --- | --- | --- | --- | --- | --- |
|  |  |  |  |  |  |
|  |  |  |  |  |  |
| Headline Type | 0.539 |  | *0.603, 1.476* |  | *0.079, 0.113* |
|  |  |  |  |  |  |
| Phase | 0.101 |  | *0.289* |  | *0.567* |
|  |  |  |  |  |  |
| Headline Type × Phase | 0.177 |  | *0.028, 0.124* |  | *0.167, 0.394* |
|  |  |  |  |  |  |

*Note.* The column Sensitivity reports the smallest beta coefficients that could be detected with 80% for each of the effects of interest. The columns Experiment 1 and Experiment 2 report the range of coefficients empirically observed as (*minimum, maximum)*.

**Table S3**

Summary of Sensitivity Analyses for Veracity Ratings Conditionalized on Correction Recognition

| Effect | Sensitivity |  | Experiment 1 |  | Experiment 2 |
| --- | --- | --- | --- | --- | --- |
|  |  |  |  |  |  |
|  |  |  |  |  |  |
| Headline Type | 0.058 |  | *0.082, 0.192* |  | *0.059, 0.161* |
|  |  |  |  |  |  |
| Phase | 0.077 |  | *0.345, 0.615* |  | *0.223, 0.659* |
|  |  |  |  |  |  |
| Correction Recognition | 0.114 |  | *0.394, 0.699* |  | *0.432, 0.831* |
|  |  |  |  |  |  |
| Headline Type × Phase | 0.105 |  | *0.052, 0.402* |  | *0.038, 0.392* |
|  |  |  |  |  |  |
| Headline Type × Correction Recognition | 0.124 |  | *0.050, 0.261* |  | *0.058, 0.271* |
|  |  |  |  |  |  |
| Phase × Correction Recognition | 0.119 |  | *0.212, 0.959* |  | *0.123, 0.979* |
|  |  |  |  |  |  |
| Headline Type × Phase × Correction Recognition | 0.276 |  | *0.001, 0.298* |  | *0.003, 0.265* |
|  |  |  |  |  |  |

*Note.* The column Sensitivity reports the smallest beta coefficients that could be detected with 80% for each of the effects of interest. The columns Experiment 1 and Experiment 2 report the range of coefficients empirically observed as (*minimum, maximum)*.

**Figure S1**

*Sensitivity Analyses for Veracity Ratings*

*Note.* The dashed red line indicates 80% power. The black line depicts the increase in statistical power (y-axis) as the effect size increases (x-axis).

**Figure S2**

*Sensitivity Analyses for Correction Recognition*

*Note.* The dashed red line indicates 80% power. The black line depicts the increase in statistical power (y-axis) as the effect size increases (x-axis).

**Figure S3**

*Sensitivity Analyses for* *Veracity Ratings Conditionalized on Correction Recognition*

*Note.* The dashed red line indicates 80% power. The black line depicts the increase in statistical power (y-axis) as the effect size increases (x-axis).

2. **Instructional Comprehension Accuracy Check**

Every instruction screen was followed by an assessment of how well participants understood the instructions. There were four questions in session 1: one for Phase 1, two for Phase 2, and 1 for Phase 3. There was one comprehension question in session 2 for Phase 4. Table S4 shows participants’ total accuracy for the five comprehension questions.

**Table S4**

Participant Accuracy on Instruction Comprehension Questions

| Number Correct (out of 5) | Experiment 1 |  | Experiment 2 |
| --- | --- | --- | --- |
|  |  |  |  |
|  |  |  |  |
| 5 / 5 | 239 |  | 261 |
|  |  |  |  |
| 4 / 5 | 63 |  | 78 |
|  |  |  |  |
| 3 / 5 | 34 |  | 21 |
|  |  |  |  |
| 2 / 5 | 11 |  | 7 |
|  |  |  |  |
| 1 / 5 | 4 |  | 2 |
|  |  |  |  |

**References**

Kumle, L., Võ, M. L.-H., & Draschkow, D. (2021). Estimating power in (generalized) linear mixed models: An open introduction and tutorial in R. *Behavior Research Methods*, *53*(6), 2528–2543. <https://doi.org/10.3758/s13428-021-01546-0>

1. Sensitivity was not determined separately for Experiment 2, on the basis that Experiment 2 had the same design as Experiment 1 but obtained a slightly larger sample size and lower variance. Sensitivity was therefore necessarily similar (if slightly *better)* for Experiment 2 compared to Experiment 1.
